# Genome sequencing of turmeric provides evolutionary insights into its medicinal properties

**DOI:** 10.1038/s42003-021-02720-y

**Published:** 2021-10-15

**Authors:** Abhisek Chakraborty, Shruti Mahajan, Shubham K. Jaiswal, Vineet K. Sharma

**Affiliations:** MetaBioSys Group, Department of Biological Sciences, Indian Institute of Science Education and Research Bhopal, Bhopal, India

**Keywords:** Plant sciences, Computational biology and bioinformatics

## Abstract

*Curcuma longa*, or turmeric, is traditionally known for its immense medicinal properties and has diverse therapeutic applications. However, the absence of a reference genome sequence is a limiting factor in understanding the genomic basis of the origin of its medicinal properties. In this study, we present the draft genome sequence of *C. longa*, belonging to Zingiberaceae plant family, constructed using 10x Genomics linked reads and Oxford Nanopore long reads. For comprehensive gene set prediction and for insights into its gene expression, transcriptome sequencing of leaf tissue was also performed. The draft genome assembly had a size of 1.02 Gbp with ~70% repetitive sequences, and contained 50,401 coding gene sequences. The phylogenetic position of *C. longa* was resolved through a comprehensive genome-wide analysis including 16 other plant species. Using 5,388 orthogroups, the comparative evolutionary analysis performed across 17 species including *C. longa* revealed evolution in genes associated with secondary metabolism, plant phytohormones signaling, and various biotic and abiotic stress tolerance responses. These mechanisms are crucial for perennial and rhizomatous plants such as *C. longa* for defense and environmental stress tolerance via production of secondary metabolites, which are associated with the wide range of medicinal properties in *C. longa*.

## Abstract (web summary)

Chakraborty et al. present the draft genome of turmeric and transcriptome of leaf tissue. Phylogenetic and comparative evolutionary analyses show evolution in genes associated with secondary metabolism, plant phytohormones signaling, and various biotic and abiotic stress tolerance responses.

## Introduction

Turmeric, a common name for *C. longa*, has been traditionally used as a herb and spice for 4000 years in Southern Asia^1^. It has a long history of usage in medicinal applications, as an edible dye, as a preservative in many food materials, in religious ceremonies, and is now widely used in cosmetics throughout the world^1,2^. It is a perennial rhizomatous monocot herbal plant of the *Curcuma* genus comprising of more than 130 known species affiliated with the family of Zingiberaceae comprising more than 1300 species, which are widely distributed in tropical Africa, Asia, and America^1,3^. The Zingiberaceae family is enriched in rhizomatous and aromatic plants that produce a variety of bioactive compounds such as curcumin. The association of endophytes with Zingiberaceae family plants helps in enhanced production of various secondary metabolites that further confer medicinal properties to species such as *C. longa*^3^.

Secondary metabolism is one of the key adaptations in plants to cope with the environmental conditions through production of a wide range of common or plant-specific secondary metabolites^4,5^. These secondary metabolites also play a key role in plant defense mechanisms, and several of these metabolites have numerous pharmacological applications in herbal medicines and phytotherapy^6^. The pathways for biosynthesis of various secondary metabolites including phenylpropanoids, flavonoids (such as curcuminoids), terpenoids, and alkaloids are found in *C. longa*^7–10^. Other constituents such as volatile oils, proteins, resins, and sugars are also present in *C. longa*^10^. Flavonoids are known to have anti-inflammatory, antioxidant, and anti-cancer activities^11^. Phenylpropanoids are also of great importance because of their antioxidant, anti-cancer, anti-microbial, anti-inflammatory, and wound-healing activities^12^. The other class of secondary metabolites, terpenoids, are also known to possess anti-cancer and anti-malarial properties^13^.

The three curcuminoids namely curcumin, demethoxycurcumin, and bisdemethoxycurcumin, are responsible for the yellow color of turmeric^10^. Among these, the primary bioactive component of turmeric is curcumin, which is a polyphenol-derived flavonoid compound and also known as diferuloylmethane^10^. Curcumin shows broad-spectrum antimicrobial properties against bacteria, fungi and viruses^14^, and also possesses anti-diabetic, anti-inflammatory, antifertility, anti-coagulant, hepatoprotective, and hypertension protective properties^10,15^. Being an excellent scavenger of reactive oxygen species (ROS) and reactive nitrogen species, its antioxidant activity also controls DNA damage by lipid peroxidation mediated by free-radicals, and thus provides it with anti-carcinogenic properties^16^. Due to these medicinal properties, turmeric has been of interest for scientists from many decades. Notably, the major bioactive compound of turmeric i.e., curcumin, is being recognized as “Pan-assay interference compounds” (PAINS), and “Invalid metabolic panaceas” (IMPS) candidate^17–19^. As a PAINS candidate, curcumin can result in false-positive assay readouts, which are not the actual results of the interactions with other compounds in the assay but artefacts. Similarly, another report considered curcumin as a poor drug lead and IMPS candidate because of its promiscuous bioactivity and metabolic instability^17^. However, recently, the efficiency of curcumin as a drug lead was improved through prodrug-based curcumin nanoparticles generation that increased its chemical stability, and reduced its aggregation^20^.

Several studies have been carried out to study the secondary metabolites and medicinal properties of this plant^21,22^. Recently, the transcriptome profiling and analysis of *C. longa* using rhizome samples have been carried out to identify the secondary metabolite pathways and associated transcripts^8,13,23,24^. However, its reference genome sequence is not yet available, which is much needed to understand the genomic and molecular basis of evolution of the unique characteristics of *C. longa*. According to the Plant DNA c-values database^25^, *C. longa* genome has an estimated size of 1.33 Gbp with 2n = 63 chromosomes, but a wide range of genome size variation (4C values ranging from 4.30 to 8.84 pg) and chromosome number variation (2n = 48 to 2n = 64) in *C. longa* was suggested^26^. Recent studies showed evidence for a ploidy level of 3X (2n = 63 chromosomes, basic chromosome number X = 21)^27,28^.

Therefore, we performed the draft genome sequencing and assembly of *C. longa* using Oxford Nanopore long reads and 10x Genomics linked reads generated on Illumina platform. The transcriptome of rhizome tissue for this plant has been known from several previous studies^8,13,23,24^, and one study also reported the transcriptome of leaf tissue^29^. Here, we carried out an extensive transcriptome sequencing of leaf tissue followed by a comprehensive transcriptome analysis, which also helped in the gene set construction. We also constructed a genome-wide phylogeny of *C. longa* with other available monocot genomes. The comparative analysis of *C. longa* with other monocot genomes revealed adaptive evolution in genes associated with plant defense and secondary metabolism, and provided genomic insights into the medicinal properties of this species.

## Results

### Sequencing of genome and transcriptome

A total of 94.8 Gbp of 10x Genomics linked read data, 47.2 Gbp Oxford Nanopore long-read data, and 32.4 Gbp of RNA-Seq data was generated from leaf tissue (Supplementary Tables 1–2). The total genomic data corresponded to ~82.4X coverage of 10x Genomics linked read data, and ~41X coverage of Nanopore long read data based on the estimated genome size of 1.15 Gbp using SGA-preqc^30^. To carry out the genome annotation, de novo transcriptome assembly was performed using RNA-Seq data from this study. All the paired-end RNA-Seq reads were trimmed and quality filtered using Trimmomatic v0.38 and used for de novo transcriptome assembly. The detailed workflow for genome and transcriptome analysis is shown in Supplementary Fig. 1.

### Assembly of the *C. longa* genome and transcriptome sequence

The genome size of *C. longa* was estimated to be 1.15 Gbp using SGA-preqc^30^ with barcode-filtered 10x Genomics linked reads, which is close to the previously estimated genome size of 1.33 Gbp^25^. *C. longa* genome was estimated to contain 4.83% heterozygosity. The *C. longa* genome sequence, assembled using Supernova v2.1.1^31^ and Flye v2.4.2^32^ had the N50 values of 15.8 Kbp and 60.9 Kbp, respectively. After correction of mis-assemblies, scaffolding, gap-closing and polishing, the final draft genome assembly (contigs with length of ≥3000 bp after scaffolding) of *C. longa* had the total size of 1.02 Gbp that comprised of 22,470 contigs with N50 value of 100.6 Kbp and GC-content of 38.75% (Supplementary Table 3). BUSCO (Benchmarking Universal Single-Copy Orthologs) completeness for Supernova assembled genome was 75.9% (Complete BUSCOs) and Flye assembled genome was 71.5% (Complete BUSCOs), which was improved to 92.4% (Complete BUSCOs) in the final polished draft *C. longa* genome assembly (Supplementary Table 4). Further, 98.9% 10x Genomics linked reads, 92.4% Nanopore long-reads, and 92.9% RNA-Seq reads were mapped on this final genome assembly. LAI value of *C. longa* genome (≥35 Kbp) that covered 72.2% of the estimated genome size was calculated as 10.26 (Supplementary Table 5) (see Methods). The genome of *C. longa* was predicted as triploid since at the variable sites, both the distributions of base frequencies showed the smallest Δlog-likelihood value for the triploid fixed model, before and after denoising (see “Methods”)^33^ (Supplementary Fig. 2a). Also, heterozygous k-mer pair coverage pattern distribution using Smudgeplot^34^ (see “Methods”) showed that 83% of the k-mer pairs corroborated to total coverage of k-mer pair = 3n and normalized minor k-mer coverage = 1/3, and thus the genome was inferred as triploid (Supplementary Fig. 2b).

The de novo transcriptome assembly of *C. longa* (from this study) using Trinity v2.9.1^35^ had a total size of 86,158,097 bp, with a total of 84,520 predicted transcripts corresponding to 36,510 genes. The complete assembly had an N50 value of 1086 bp, an average transcript length of 1019 bp and GC-content of 45.45% (Supplementary Table 6). A total of 30,552 unigenes were identified after clustering using CD-HIT-EST v4.8.1^36^ to remove the redundant gene sequences. The coding sequence (CDS) prediction from these unigenes using TransDecoder v5.5.0 resulted in 23,943 coding genes.

### Genome annotation and gene set construction

For repeat identification, a de novo custom repeat library was constructed using the final polished *C. longa* genome by RepeatModeler v2.0.1^37^, which resulted in a total of 2430 repeat families. The repeat families were clustered into 1977 representative sequences. These were used to soft-mask the genome assembly using RepeatMasker v4.1.0, which predicted 64.16% of *C. longa* genome as repetitive sequences, of which 62.37% was identified as interspersed repeats (31.61% unclassified, 28.50% retroelements and 2.26% DNA transposons). Retroelements consisted of 27.37% LTR (long terminal repeat) elements (17.19% Ty1/Copia and 9.42% Gypsy/DIRS1 elements) (Supplementary Table 7). Additionally, 7.64% of *C. longa* genome was identified as simple repeats using TRF v4.09^38^. Thus, ~70% of the genome was predicted to be constituted of simple and interspersed repetitive sequences. Among the non-coding RNAs, 1826 standard amino acid specific tRNAs (transfer RNAs) and 335 hairpin miRNAs (micro RNAs) were predicted in *C. longa* genome.

MAKER genome annotation pipeline^39^ and TransDecoder v5.5.0 predicted a total of 57,486 and 23,943 coding sequences from genome and transcriptome assemblies, respectively. Length-based filtering criteria (≥150 bp) of the above two coding gene sets resulted in 56,043 and 23,943 coding sequences from genome and transcriptome assemblies, respectively. MAKER derived filtered coding sequences were further clustered at 95% sequence identity, which resulted in 45,307 non-redundant coding sequences. These two coding gene sets were merged using BLASTN, resulting in the final *C. longa* gene set comprising of 50,401 coding gene sequences. BUSCO analysis revealed presence of 85.8% BUSCO genes (complete + fragmented) in this coding gene set of *C. longa*. Among these 50,401 genes, a total of 45,479 genes, 36,724 genes and 33,338 genes were mapped to NCBI (nr), Swiss-Prot, and Pfam-A (v32.0) databases, respectively. A total of 45,576 genes (90.43%) could be annotated against any of these three databases, and 4825 (9.57%) genes remained unannotated. Further investigations of these coding gene sequences revealed 56,936 variable nucleotide sites out of total 53,278,136 bases (0.11%) in the coding gene set. A total of 9951 out of 50,401 coding genes showed sequence variation.

### Resolving the phylogenetic position of *C. longa*

From the selected 17 plant species, a total of 104,746 orthogroups were identified using protein sequences by OrthoFinder v2.3.9^40^. Among these orthogroups, 5388 contained protein sequences from all 17 species, and were used for evolutionary analysis. Further, KinFin v1.0^41^ predicted a total of 1053 fuzzy one-to-one orthogroups containing protein sequences from all 17 selected species, which were used to construct the maximum likelihood-based phylogenetic tree of *C. longa* with 15 other monocot species and *Arabidopsis thaliana* as an outgroup.

**Fig. 1:**
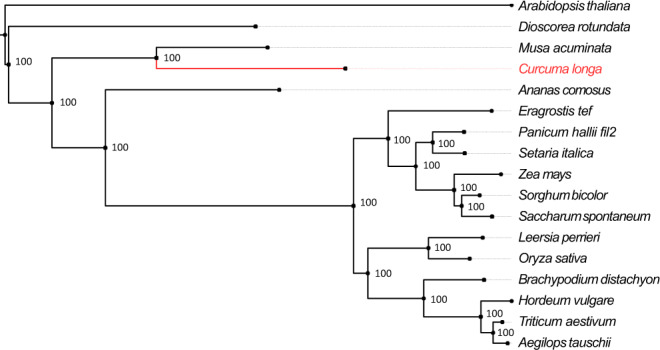
Phylogenetic position  of *C. longa*. Phylogenetic tree of *C. longa* with 15 other selected species and *Arabidopsis thaliana* as an outgroup species. The values mentioned at the nodes correspond to the bootstrap values.

All 1053 fuzzy one-to-one orthogroups were aligned, concatenated and filtered for undetermined or missing values. This filtered alignment data consisting of 851,765 alignment positions was used to construct the maximum likelihood-based species phylogenetic tree of *C. longa* with 15 other monocot species available on Ensembl plants release 47 and *Arabidopsis thaliana* as the outgroup (Fig. 1). Position of *C. longa* and the other selected monocots in this genome-wide phylogeny were supported by previously reported phylogenies^42–45^. In our phylogeny, *Ananas comosus* showed an earlier divergence among the monocots from Poales order, which was also supported by previously reported studies^42,45^. Among all selected monocot species in our study, species from Dioscoreales order showed the earliest divergence, supported by the previously reported studies^44,45^. From our genome-wide phylogeny constructed using the selected monocots, it is apparent that *Musa acuminata* was comparatively closer to *C. longa*. Belonging to the same phylogenetic order Zingiberales, *C. longa* and *Musa acuminata* shared the same clade in the species phylogenetic tree. Species from the Zingiberales order are closer related to species from the Poales order in comparison to the species from the Dioscoreales order.

### Genes with signatures of adaptive evolution

Genes with site-specific signatures of adaptive evolution were identified in *C. longa*. 3230 genes showed unique amino acid substitution with respect to the other selected species. Among these 3230 genes, 2429 genes were identified to have functional impacts using sorting intolerant from tolerant (SIFT), and were considered further. Further, 569 genes were found to contain positively selected codon sites with greater than 95% probability. In addition to these site-specific signatures of evolution, 63 genes showed higher rate of nucleotide divergence, and 306 genes showed positive selection in *C. longa* with FDR (false discovery rate)-corrected *p*-values < 0.05. These positively selected genes had positively selected codon sites with greater than 95% probability. A total of 188 genes were identified containing more than one of the signatures of adaptive evolution namely positive selection, unique amino acid substitution with functional impact and higher rate of nucleotide divergence.

**Fig. 2:**
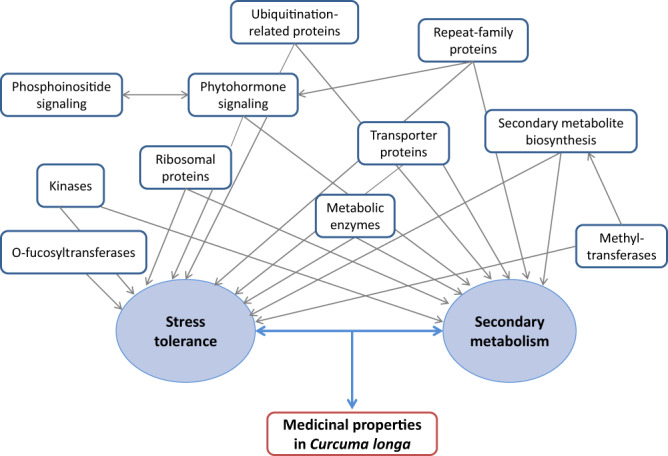
Functional categories of MSA genes. Functional association of MSA genes with medicinal properties of *C. longa*.

The positively selected genes, genes with higher nucleotide divergence, genes showing site-specific evolutionary signatures, and MSA (multiple signs of adaptive evolution) genes were mapped on KEGG (Kyoto encyclopedia of genes and genomes) pathways, and classified in eggNOG COG (clusters of orthologous groups) categories and GO (Gene ontology) enrichment categories (Supplementary Tables 8–20). A total of 172 out of 188 MSA genes (Supplementary Data 1) were associated with categories essential for plant secondary metabolism and defense responses against environmental stress conditions i.e., biotic stress and abiotic stress (Fig. 2).

### Adaptive evolution of plant defense associated genes

Several genes known to provide plant immunity against pathogen infection or disease were found in MSA genes. Since plants lack adaptive immune response, the innate immune response in plants is provided by PAMP-triggered (PTI) and effector-triggered (ETI) immunity with the help of two plant stress hormones, salicylic acid (SA) and jasmonic acid (JA) signaling pathway^46^. Among these MSA genes, *JAR1* (Jasmonate resistant 1) is required for conversion of jasmonic acid to its bioactive form jasmonoyl-L-isoleucine (JA-Ile), *COI1* (Coronatine-insensitive 1) is a receptor of JA-Ile and thus regulates downstream JA-signaling processes, *MPK9* (MAP kinase 9) gene expression is regulated by JA as well as SA treatments and is involved in PAMP-triggered immunity, *BSK1* (Brassinosteroid-signaling kinase 1) plays an important role in brassinosteroid signaling pathway that is involved in plant innate immunity and also has an antagonistic relationship with JA signaling effects^47–49^. Also, *WRKY* transcription factor is induced by pathogen attack and is involved in SA-signaling pathway mediated plant immunity, *MYB48* (Myeloblastosis 48) transcription factor that is involved in salicylic acid-mediated response negatively regulates effector-triggered immunity, and *EIN3* (ethylene-insensitive 3) negatively regulates SA levels and PAMP-triggered plant innate immunity^50–52^. Jasmonic acid and salicylic acid elicit the production and accumulation of secondary metabolites such as phenolics, terpenoids, alkaloids, and glycosides, in medicinal plants^53^.

Three O-fucosyltransferase family proteins—*AT1G22460, AT3G11540*, and *AT4G08810* were found in MSA genes category, and are involved in plant immunity. Previously it has been shown that the lack of fucosylation of genes led to increased disease susceptibility in *Arabidopsis sp*. by affecting PTI, ETI as well as stomatal and apoplastic defense^54^. Three ubiquitin-conjugating enzymes—*UBE2E* (ubiquitin-conjugating enzyme E2 E), *UBE2D* (ubiquitin-conjugating enzyme E2 D), *AT2G16920* and six E3 ubiquitin-protein ligase genes—*COP1* (constitutive photomorphogenic protein 1)*, AT1G55250, AT4G27880, AT4G28370, AT3G26730*, and *AT5G45360* also showed multiple signs of adaptive evolution. Ubiquitination-related proteins affect hypersensitive-response (HR) and phytohormone signaling mediated pathogen defense by targeting proteins for proteasomal degradation^55,56^. These ubiquitin ligase proteins also regulate various abiotic stress responses e.g., drought, salinity, temperature^56^. Among the MSA genes, three ribosomal subunit proteins are regulated by signaling molecules such as methyl jasmonate, salicylic acid and environmental stress e.g., cold, heat, ultraviolet (UV), drought, salinity^57,58^. Two cell cycle related MSA genes—cyclin-A and *APC10* (anaphase promoting complex subunit 10) are involved in plant immunity, disease resistance as well as abiotic stress responses^59,60^. 19 different repeat family genes belonging to WD-family repeats, leucine-rich repeats (LRR), pentatrico peptide repeats (PPR), tetratrico peptide repeat (TPR)-like superfamily repeats, armadillo (ARM) superfamily repeats and ankyrin repeats were identified as MSA genes. These repeat family proteins are involved in plant innate immunity as well as abiotic stress tolerance^61,62^.

### Abundance of secondary metabolism pathways in *C. longa* genome

Genes associated with secondary metabolite biosynthesis and secondary metabolism were found to be abundant (104 out of 188 genes) among the MSA genes in *C. longa*. Of these, *DWF4* (Dwarf 4) is involved in brassinosteroid biosynthesis, *CHI* (chalcone isomerase) plays a key role in anthocyanin biosynthesis, *ADT* (arogenate dehydratase) has a role in lignin biosynthesis, *GST* (glutathione S-transferase) aids in glucosinolate biosynthesis^63–66^. Shikimate kinase, which is a central enzyme involved in shikimic acid pathway required for production of a wide range of phenolic group of secondary metabolites^67^, was identified as an MSA gene. Methyltransferases e.g., *AT1G04430*, serine hydroxymethyltransferase, lysine methyltransferase-like, Protein arginine methyltransferase are also involved in secondary metabolites biosynthesis and responsible for methylation of secondary metabolites, which in turn assist in disease resistance and stress tolerance in plants^68,69^. These secondary metabolites possess various medicinal applications such as anti-inflammatory, antimicrobial, antioxidant, and anti-carcinogenic properties^6,12,70^. The functional role of selected MSA genes involved in secondary metabolite biosynthesis and medicinal properties of the resultant metabolites are mentioned in Supplementary Table 21. Transporter genes e.g., three ATP-binding cassette proteins, *KT1* (K^+^ Transporter 1) and *KEA3* (K^+^ Efflux Antiporter 3) potassium transporters, *NRT* (nitrate transporter), *SULTR2;1* (sulfate transporter 2;1) also showed multiple signs of adaptation. The evolution of these transporter genes appears important since the alteration in concentration of metal ions regulate accumulation and translocation of secondary metabolites^71–73^.

**Fig. 3:**
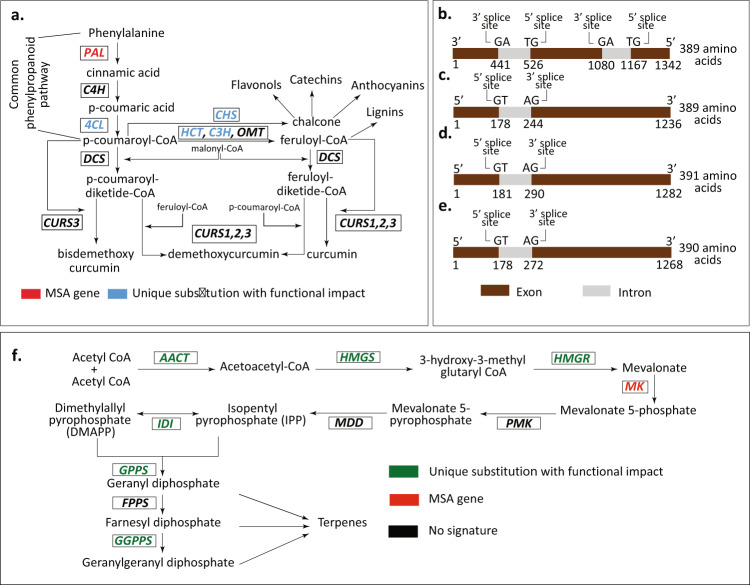
Pathways and genes involved in secondary metabolism of *C. longa*. **a** Curcuminoid biosynthesis pathway in *C. longa*^75,76,79^. *PAL* = phenylalanine ammonia lyase, *C4H* = cinnamate-4-hydroxylase, *4CL* = 4-coumarate-CoA ligase, *HCT* = hydroxycinnamoyl transferase, *C3H* = cinnamate-3-hydroxylase, *OMT* = O-methyltransferase, *CHS* = chalcone synthase, *DCS* = diketide-CoA synthase, *CURS1* = curcumin synthase 1, *CURS2* = curcumin synthase 2, *CURS3* = curcumin synthase 3. **b** Structure of *DCS* gene. **c** Structure of *CURS1* gene. **d** Structure of *CURS2* gene. **e** Structure of *CURS3* gene. **f** Evolutionary signatures in mevalonate pathway of terpenoid biosynthesis^80^. *AACT* = acetoacetyl-CoA thiolase, *HMGS* = HMG-CoA synthase, *HMGR* = HMG-CoA reductase, *MK* = mevalonate kinase, *PMK* = phosphomevalonate kinase, *MDD* = mevalonate-5-diphosphate decarboxylase, *IDI* = isopentenyl diphosphate isomerase, *GPPS* = geranyl diphosphate synthase, *FPPS* = farnesyl diphosphate synthase, *GGPPS* = geranylgeranyl diphosphate synthase.

Polyketides, such as curcuminoids, represent a diverse class of secondary metabolites, which are crucial for plants survival under environmental challenges^74^. Curcuminoid biosynthesis pathway is the most important secondary metabolism pathway in *C. longa* where phenylalanine is converted to coumaroyl-CoA via cinnamic acid and coumaric acid^75^. Conversion of phenylalanine to coumaroyl-CoA is also part of phenylpropanoid biosynthesis pathway^76^. The two key enzymes *PAL* (phenylalanine ammonia lyase) and *4CL* (4-coumarate-CoA ligase), in this step showed evolutionary signatures. *4CL* gene possessed unique amino acid substitution with functional impact. Notably, the *PAL* gene, which is involved in conversion of phenylalanine to cinnamic acid in the curcuminoid biosynthesis pathway^75^, showed positive selection and unique amino acid substitution in this study, and thus was identified as an MSA gene. Also, the *C3H* (cinnamate-3-hydroxylase) and *HCT* (hydroxycinnamoyl transferase) genes that are involved in conversion of coumaroyl-CoA to feruloyl-CoA showed unique amino acid substitution with functional impact (Fig. 3a).

Further, coumaroyl-CoA and feruloyl-CoA are used for the production of curcumin, demethoxycurcumin, and bisdemethoxycurcumin via coumaroyl-diketide-CoA and feruloyl-diketide-CoA, catalyzed by four enzymes—*CURS1* (curcumin synthase 1)*, CURS2* (curcumin synthase 2)*, CURS3* (curcumin synthase 3), and *DCS* (diketide-CoA synthase)^75^. To identify these enzymes in the genome and transcriptome assemblies constructed in this study, we mapped the coding gene sequences of *CURS1, CURS2, CURS3*, and *DCS* genes on these assemblies. All four enzymes were found to be present in the de novo genome assembly, in the gene set derived from MAKER pipeline, and in the gene set derived from de novo transcriptome assembly of *C. longa*. Using Exonerate, we further constructed the gene structures for these four major curcuminoid biosynthesis genes (Fig. 3b–e). Each of the *CURS1, CURS2* and *CURS3* genes consisted of two exons and one intron. *DCS* gene consisted of three exons and two introns. *DCS* and *CURS* genes are members of chalcone synthase (*CHS*) family^77^, and the genes from *CHS* family generally consist of two exons and one intron^78^, which is consistent with previous studies and also further supported by our findings.

Coumaroyl-CoA is also a precursor for biosynthesis of anthocyanins, flavonols and catechins^76^. A key enzyme *CHS*, responsible for conversion of coumaroyl-CoA to chalcone, showed unique substitution with functional impact (Fig. 3a). The *FLS* (flavonol synthase) gene, required for flavonols synthesis^76^, possessed unique amino acid substitution. Another intermediate of curcuminoid biosynthesis pathway, feruloyl-CoA, is a precursor for lignin biosynthesis^79^. Enzymes involved in production of lignins from feruloyl-CoA also showed signatures of evolution. *CAD* (cinnamyl alcohol dehydrogenase) was positively selected, *PRX* (peroxidase) and *CCR* (cinnamoyl-CoA reductase) exhibited unique substitution with functional impact, and *LAC* (laccase) showed both unique amino acid substitution and positively selected codon site.

Terpenoid biosynthesis pathway also showed distinct evolutionary signatures in *C. longa*. 7 out of 10 enzymes in the mevalonate pathway of terpenoid backbone biosynthesis^80^ were found to be evolved in comparison to the other selected species (Fig. 3f). Among these, *AACT* (acetoacetyl-CoA thiolase), *HMGS* (HMG-CoA synthase), *HMGR* (HMG-CoA reductase), *IDI* (isopentenyl diphosphate isomerase), *GPPS* (geranyl diphosphate synthase)*, GGPPS* (geranylgeranyl diphosphate synthase) showed unique amino acid substitutions with functional impact. *MK* (mevalonate kinase) exhibited both unique substitution and positive selection, and thus was found among the MSA genes.

### Evolution of curcuminoid biosynthesis pathway

**Fig. 4:**
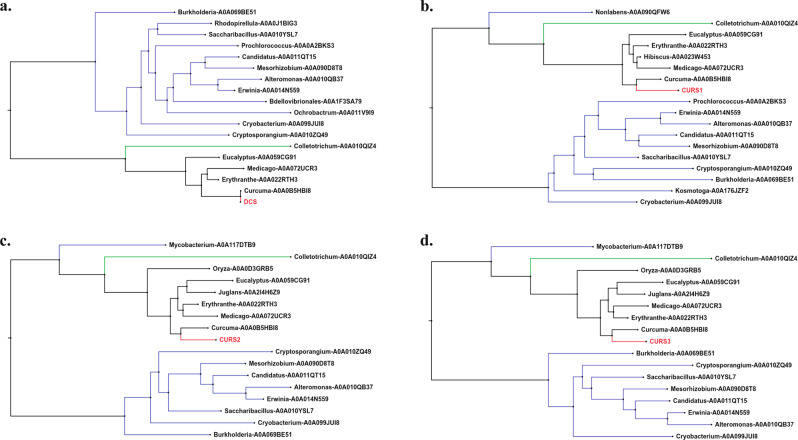
Phylogenetic relationships of candidate enzymes of lower curcuminoid biosynthesis pathway in *C. longa* with their distant orthologous genes. **a** *DCS* gene. **b** *CURS1* gene. **c** *CURS2* gene. **d** *CURS3* gene. Blue colored line denotes Bacterial orthologs, dark green colored line denotes fungal orthologs, black colored line denotes angiosperm orthologs, and red colored line denotes the genes of interest in *C. longa*.

The ten enzymes involved in curcuminoid biosynthesis pathway were analyzed to elucidate their origin by remote homology finding and to examine their phylogenetic position with respect to the genes from other plant species (see “Methods”). These enzymes were identified in the coding gene set of *C. longa* using CAPS_protocol^81^. Among the ten enzymes, *HCT* had all the phylogenetically closer orthologs (transferases) from angiosperm plant division. However, *C4H* (cinnamate-4-hydroxylase) had a Bryophyte ortholog and *C3H* had a Gymnosperm ortholog, along with other angiosperm orthologs (monooxygenase and oxidoreductase) (Supplementary Fig. 3). The remaining seven enzymes had ancestor genes from bacterial origin, and fungal orthologs were also observed in the case of six of these enzymes. Identified orthologs of *PAL*, *4CL,* and *OMT* (O-methyltransferase) enzymes (Supplementary Fig. 3) show ammonia-lyase activity, catalytic activity, and methyltransferase activity, respectively. The four key enzymes unique to curcuminoid biosynthesis pathway i.e., *DCS*, *CURS1*, *CURS2,* and *CURS3* (Type III polyketide synthases) had similar bacterial and fungal orthologs (Fig. 4a–d) that were annotated as 3-oxoacyl-ACP synthase, and chalcone and stilbene synthase, respectively. 3-oxoacyl-ACP synthase plays a role in fatty acid biosynthesis in bacteria^82^, and chalcone and stilbene synthase that was identified as a fungal ortholog is a polyketide synthase and a key enzyme involved in secondary metabolite biosynthesis pathways^83^.

Gene family evolution analysis (using CAFÉ v4.2.1)^84^ for these ten enzymes showed that gene families of 8 out of 10 enzymes were expanded in *C. longa* compared to its immediate ascending node, and gene families of *HCT* and *OMT* genes were contracted (Supplementary Table 22). Among these, *4CL* gene family showed comparatively more expansion in terms of gene numbers. *DCS*, *CURS1*, *CURS2*, and *CURS3* genes were identified as members of the same gene family, which underwent expansion in the CAFÉ analysis.

## Discussion

*C. longa* is a monocot species from Zingiberaceae plant family and is widely known for its medicinal properties and therapeutic applications^15^. In this study, we carried out the whole-genome sequencing and reported the draft genome sequence of *C. longa*. This is the first whole-genome sequenced and analyzed from Zingiberaceae plant family to the best of our knowledge, which comprises of more than 1300 species, and thus will act as a valuable reference for studying the members of this family including those of *Curcuma* genus. Genomic polyploidy in members of *Curcuma* genus is well known from previously reported experimental studies^27,85,86^. In this study, we estimated the ploidy level of *C. longa* genome using next-generation sequencing (NGS) reads, and showed the triploid nature of *C. longa* genome, which is also supported by the previous experimental studies^27,28,85,86^. The application of Oxford Nanopore long-reads and 10x Genomics linked read sequencing that has the potential to resolve complex polyploid genomes^87^, helped in successfully constructing the *C. longa* draft genome of 1.02 Gbp with a decent N50 of 100.6 Kbp. After assembly correction, scaffolding, gap-closing and polishing, the BUSCO completeness of final *C. longa* genome improved to 92.4%, which is similar to other plant genomes, thus indicating the usefulness of post-assembly processing^88^. It is noteworthy that the LAI value of *C. longa* genome (≥35 Kbp) was estimated at 10.26, which also corresponds to a reference quality genome assembly^89^.

Since the construction of a comprehensive gene set was essential to explore the genetic basis of its medicinal properties, both genome and transcriptome assemblies, and an integrated approach using de novo and homology-based methods were used, which resulted in the final set of 50,401 genes. The identification of all Type III polyketide synthase genes *CURS1, CURS2, CURS3*, and *DCS*, involved in the biosynthesis of the three most important secondary metabolites (curcuminoids)—curcumin, demethoxycurcumin and bisdemethoxycurcumin, in both genome and transcriptome assemblies also attests to the quality and comprehensiveness of our genome and transcriptome assembly. Further, the revelation of complete gene structures of the above four biosynthesis genes of curcuminoid pathway from the draft genome of this plant is likely to help further studies and improve commercial exploitation of these curcuminoids that find wide applications as coloring agents, food additives and possess antioxidant, anti-inflammatory, anti-microbial, neuroprotective, anti-cancer, and many other medicinal properties^90^.

Repetitive sequence prediction revealed that ~70% of the genome consisted of repeat elements, which is similar to other plant genome, such as *Triticum urartu*^91^. Notably among the LTR repeat elements, Ty1/Copia elements (17.19%) were more abundant than Gypsy/DIRS1 elements (9.42%), which corroborates with the observations made in the case of *Musa acuminata* species from the same Zingiberales plant order, and thus appears to be a specific signature of repeat elements in Zingiberales order^92^.

The genome-wide phylogenetic analysis of *C. longa* with 15 other representative monocot species available on Ensembl plants revealed the relative position of *C. longa*, which was supported by previously reported phylogenies using 1685 gene partitions, and using phytocystatin gene *CypCl*^42,43^. Ren et al. also showed similar phylogenetic position of *C. longa* with other selected monocots – *Dioscorea sp*., *Musa acuminata*, *Brachypodium distachyon*, *Oryza sativa*, *Panicum sp*., *Setaria italica*, *Zea mays*, *Sorghum bicolor* using genome and transcriptome data of 105 angiosperms^44^. Also, an updated megaphylogeny for vascular plants showed similar relative phylogenetic position of *C. longa* with respect to the selected monocots^45^. Further, selected species from Poales order also showed similar relative positions with respect to each other^45^. Absence of any polytomy in the phylogenetic tree is because of large number of genomic loci and a high bootstrap value, or no multiple speciation events took place at the same time. Taken together, the genome-wide phylogenetic analysis of *C. longa* confirmed its phylogenetic position and will be a useful reference for further studies.

Analysis of genes with signatures of adaptive evolution using 5388 orthologous gene sets revealed that a large proportion (~91%) of the genes with multiple signs of adaptive evolution (MSA) were associated with plant defense mechanisms against biotic and abiotic stress responses, and secondary metabolism. Notable ones among these are the genes associated with Jasmonic acid and salicylic acid signaling pathways. These two pathways are important components of plant innate immune response^46^, and also affect plant secondary metabolism by regulating the production of secondary metabolites^53^, thus play a crucial role in plant-pathogen interaction. Jasmonic acid is also reported to have a role in induction and growth of rhizome in vitro through its interaction with ethylene, which is important for a rhizomatous plant like *C. longa*^93^. Further, one of the genes (*PAL*) for the enzymes involved in curcuminoid biosynthesis pathway was also found to have signatures of adaptive evolution, which is an important observation because curcuminoid is the most important secondary metabolite of *C. longa*. The genes for the other four key enzymes (*CURS1*, *CURS2*, *CURS3*, *DCS*) of this pathway could not be found in the list of MSA genes since these genes are unique to *Curcuma* genus and were absent in the other species considered for the evolutionary analysis.

The gene family evolution analysis showed that gene families of all ten enzymes of curcuminoid biosynthesis pathway underwent expansion/contraction compared to the immediate ascending node of *C. longa* in the species phylogenetic tree, which suggests evolution of this pathway in *C. longa*. Further, evolutionary origin of these ten enzymes revealed that homologs for the enzymes exist in bacterial and fungal species indicating ancestral origin of these genes. Interestingly, in case of the four key enzymes (*CURS1*, *CURS2*, *CURS3*, *DCS*), the bacterial ancestor genes were involved in primary metabolism, and fungal ancestor genes (member of polyketide synthase family) were involved in secondary metabolism. Taken together, these observations suggest an evolution of these genes in *C. longa* to play key roles in curcuminoid biosynthesis pathway. Furthermore, several major secondary metabolism pathways were also found to be evolved in *C. longa* compared to the other selected plant species in this study. Also, the key enzymes involved in the biosynthesis pathways of terpenoid backbone and important compounds in phenolic group of secondary metabolites (e.g., curcuminoids, anthocyanins, lignins, phenylpropanoids) showed signatures of adaptive evolution, which is an important observation since these pathways are associated with the wide range of medicinal properties of *C. longa*.

It is important to mention here that the biosynthesis of secondary metabolites such as polyketides (curcuminoids), which are crucial for plants survival under environmental challenges, are regulated by biotic and abiotic stress responses^94,95^. Also, it is known that secondary metabolites in plants are primarily produced in response to environmental stress and for plant defense, which help in better survival under various environmental conditions^96^, and several of these secondary metabolites also possess medicinal values. This also seems to be the case with *C. longa* where the observed abundance of adaptively evolved genes associated with plant defense mechanisms and secondary metabolism makes it tempting to speculate that these genes gradually evolved for environmental adaptation and to confer resistance to a perennial rhizomatous plant like *C. longa*. Several of the metabolites produced in the above processes possess diverse medicinal properties, and thus provide *C. longa* with its medicinal characteristics and traditional significance.

## Methods

### Sample collection, library preparation, and sequencing

The plant sample was collected from an agricultural farm (23.2280252°N 77.2088987°E) located in Bhopal, Madhya Pradesh, India. The leaves were homogenized in liquid nitrogen for DNA extraction using Carlson lysis buffer. Species identification was performed by PCR (polymerase Chain Reaction) amplification of a nuclear gene (internal transcribed spacer ITS) and a chloroplast gene (Maturase K), followed by Sanger sequencing at the in-house facility. The linked read library construction from the extracted DNA was done with the help of Chromium Controller instrument (10x Genomics) using Chromium™ Genome Library & Gel Bead Kit v2 by following the manufacturer’s instructions. The Nanopore library was prepared using SQK-LSK109 kit and sequenced on MinION platform using FLO-MIN106 flow cell. Using the same plant sample, RNA extraction was carried out from the powdered leaves using TriZol reagent (Invitrogen, USA). The transcriptomic library was prepared with TruSeq Stranded Total RNA Library Preparation kit by following the manufacturer’s protocol with Ribo-Zero Workflow (Illumina, Inc., USA). The quality of 10x Genomics and transcriptomic libraries was evaluated on Agilent 2200 TapeStation using High Sensitivity D1000 ScreenTape (Agilent, Santa Clara, CA) prior to sequencing. The prepared genomic (10x Genomics) and transcriptomic libraries were sequenced on Novaseq 6000 (Illumina, Inc., USA) generating 150 bp paired-end reads. The detailed DNA and RNA extraction steps and other methodologies are mentioned in Supplementary Notes 1.

### Genomic data processing and assembly

The barcode sequences were trimmed from raw 10x Genomics linked reads using a set of python scripts (https://github.com/ucdavis-bioinformatics/proc10xG). The genome size of *C. longa* was estimated using a k-mer count distribution method implemented in SGA-preqc^30^ (Supplementary Notes 2). A total of 631.11 million raw 10x Genomics linked reads corresponding to ~82.4X coverage were used for generating a de novo assembly using Supernova assembler v2.1.1 with maxreads = all option and other defaults settings^31^. The haplotype-phased assembled genome was generated using Supernova mkoutput in ‘pseudohap’ style.

The 10x Genomics linked reads were run through Longranger basic v2.2.2 (https://support.10xgenomics.com/genome-exome/software/pipelines/latest/installation) for barcode processing and were used to detect and correct mis-assemblies in Supernova assembled genome using Tigmint v1.1.2^97^. The first round of scaffolding was carried out using ARCS v1.1.1 (default parameters) to generate more contiguous assembly using 10x Genomics linked reads^98^. Further scaffolding was performed to improve the contiguity using AGOUTI v0.3.3 with the quality filtered paired-end RNA-Seq reads from our study, which was also used in de novo transcriptome assembly^99^. Adapter-processed Nanopore long-reads (>20 Kb) were also used for scaffolding of the genome assembly using LINKS v1.8.6 with default parameters^100^.

Oxford Nanopore long-read data was base-called using Guppy v4.4.0 (Oxford Nanopore Technologies), and adapter sequences were removed using Porechop v0.2.4 (Oxford Nanopore Technologies). The adapter-processed Nanopore reads were used to perform long reads-based de novo assembly of *C. longa* genome by Flye^32^ with default parameters using the version v2.4.2 that provides better assembly coverage and contiguity. This assembly was polished using barcode-processed 10x Genomics linked reads using Pilon^101^ v1.23 in three iterations to fix local mis-assemblies, small indels, or individual base errors that could be introduced from long, error-prone Nanopore reads. Scaffolding of this polished assembly was performed with barcode-processed 10X Genomics linked reads, quality-filtered RNA-Seq reads from our study, and Nanopore long reads (>20 Kb) using ARCS v1.1.1^98^, AGOUTI v0.3.3^99^, and LINKS v1.8.6^100^, respectively.

The genome assembly of *C. longa* generated from 10x genomics linked reads and Nanopore long-reads were merged together using Quickmerge v0.3 in order to achieve a more contiguous assembly^102^. Gap-closing of this scaffolded assembly was performed with barcode-processed linked reads using Sealer v2.1.5 with k-mer value from 30 to 120 with an interval of 10 bp using a Bloom filter-based approach^103^, and LR_Gapcloser^104^ with Nanopore long-reads. Finally, the assembly quality was improved by Pilon v1.23 using barcode-processed linked reads to fix small indels, individual base errors, or local mis-assemblies that could be introduced by the previous scaffolding steps^101^. The other details about the genome assembly post-processing are mentioned in Supplementary Notes 2.

Further, in order to validate the final genome assembly of *C. longa*, barcode-removed 10x Genomics linked reads, adapter-processed Nanopore long-reads, and quality-filtered RNA-Seq reads from this study were individually mapped to the assembly using BWA-MEM^105^ (v0.7.17), Minimap2^106^ (v2.17), and HISAT2^107^ (v2.2.1) respectively, and the mapping statistics was calculated using samtools^108^ (v1.9) “flagstat”. BUSCO v5.2.1 was used to assess the genome assembly completeness using embryophyta_odb10 database^109^. Also, LTR Assembly Index (LAI) was used to assess the assembly continuity using LTR retrotransposons (LTR-RTs) that are highly abundant and difficult to assemble in repeat rich plant genomes^89^. GenomeTools^110^ v1.6.1, and LTR_retriever^111^ v2.9.0 (default parameters) was used to calculate LAI score in the final *C. longa* genome assembly.

The genome ploidy was estimated with a statistical approach using Gaussian Mixture Model (GMM), implemented in nQuire^33^. After removing the barcode sequences, 10x Genomics linked reads were mapped to the final draft genome of *C. longa* using BWA-MEM v0.7.17^105^ with default parameters, and samtools v1.9^108^ was used to generate the sorted and indexed alignments. These alignments were processed using nQuire with default parameters to extract the variable sites with the free model and the fixed models. Distribution of these base frequencies was denoised and both the distributions, before and after denoising, were used to estimate Δlog-likelihood values for the fixed models. Also, Smudgeplot^34^ v0.2.2 was used to infer the genomic ploidy of *C. longa*. First, barcode-filtered 10x Genomics linked reads were used for k-mer counting, and k-mer frequency-based histogram generation using KMC^112^ v3.1.1 with the parameters: k-mer length of 21, excluding k-mers occurring less than 1 time, maximum value of a counter of 10,000, and excluding k-mers occurring more than 10,000 times. After extracting the k-mers between an upper and lower coverage range, Smudgeplot was used for computing the k-mer pairs set, and ploidy estimation.

The heterozygosity of *C. longa* genome was analyzed in order to assess the genomic complexity, and ease of genome assembly. The k-mer frequency-based histogram that was generated using KMC v3.1.1 in the previous step was used to estimate the heterozygosity content using GenomeScope v2.0^113^ with triploid model.

### Transcriptome assembly

The RNA-Seq data from our study and from previously available transcriptome studies of *C. longa* were used for de novo transcriptome assembly^8,13,23,24,29^. Trimmomatic v0.38 was used for adapter removal and quality filtration of raw Illumina sequence data^114^ (Supplementary Notes 2). Finally, Trinity v2.9.1 was used with default parameters to perform de novo transcriptome assembly of quality-filtered paired-end and single-end reads^35^.

Further, the quality-filtered paired-end and single-end RNA-Seq reads obtained from our study were separately used for de novo transcriptome assembly using Trinity v2.9.1^35^ with strand-specific parameter and other default settings. The strand-specificity parameter “--SS_lib_type” in Trinity v2.9.1 was used since stranded RNA-Seq data aids in more accurate transcriptome assembly and gene prediction by retaining the information of the overlapping genes from which the transcript arises^115^. Only the transcripts with length ≥500 bp were retained, and assembly statistics was calculated using a Perl script available in Trinity software package. Since this transcriptome assembly was obtained for the same individual plant that was used for genomic DNA extraction as well as genome assembly, it was used for downstream coding gene prediction steps to avoid any ambiguity. The longest isoforms for all genes were extracted from the assembled transcripts. These sequences were clustered to identify the unigenes using CD-HIT-EST v4.8.1 (90% sequence identity, 8 bp seed size)^36^.

### Genome annotation

The final polished assembly (contigs with length of ≥1000 bp after scaffolding) was used for genome annotation. RepeatModeler v2.0.1 was used to generate a de novo repeat library for this genome^37^. The resultant repeat sequences were further clustered using CD-HIT-EST v4.8.1 (90% sequence identity, seed size = 8 bp)^36^. This repeat library was used to soft-mask *C. longa* genome using RepeatMasker v4.1.0 (http://www.repeatmasker.org) and was further used for gene set construction. Genome annotation was carried out using MAKER pipeline to predict the final gene models using ab initio gene prediction programs and evidence-based approaches^39^. The transcriptome assembly of *C. longa* using previously available data along with data from our study, and protein sequences of *C. longa* along with its closest species *Musa acuminata* were used as empirical evidence in MAKER pipeline. AUGUSTUS v3.2.3, BLAST and Exonerate v2.2.0 were used in MAKER pipeline for ab initio gene prediction, evidence alignments and alignments polishing, respectively^116,117^. Tandem repeat finder (TRF) v4.09 was used to detect the tandem repeats present in *C. longa* genome^38^. Additionally, miRBase database and tRNAscan-SE v2.0.5 were used for homology-based identification of miRNAs and de novo prediction of tRNAs, respectively^118,119^. The other details about genome annotation are mentioned in Supplementary Notes 3.

### Construction of gene set

The coding genes were predicted by TransDecoder v5.5.0 from the unigenes identified in transcriptome assembly obtained from our sequencing data, using Uniref90 and Pfam (v32.0) databases for homology-based searching as ORF (open reading frame) retention criteria (https://github.com/TransDecoder/TransDecoder)^120,121^. Also, the MAKER pipeline-based gene models were clustered using CD-HIT-EST v4.8.1 (95% sequence identity, 8 bp seed size)^36^. The TransDecoder pipeline derived genes (≥150 bp) were aligned against the MAKER derived gene set (≥150 bp) using BLASTN, and the genes that did not match with identity ≥50%, query coverage ≥50% and e-value 10^−9^ were added to the MAKER gene set to prepare the final gene set of *C. longa*^122,123^. The two coding gene sets were merged to consider any unique coding gene that was not predicted from de novo genome assembly using MAKER pipeline^122^.

Further, BUSCO v5.2.1 analysis was performed using embryophyta_odb10 dataset^109^, to assess the quality, and completeness of the final coding gene set. For functional annotation, this gene set was mapped against the Swiss-Prot^124^, NCBI non-redundant (nr) database using BLASTP (e-value cut-off 10^−5^), and Pfam^121^ (v32.0) database using HMMER^125^ v3.3.2 with an e-value cut-off 10^−5^.

Further, sequence variation between different alleles in the coding gene regions were analyzed in the final coding gene set using quality-filtered paired-end RNA-Seq data from this study. First, duplicate reads were identified and removed from the quality-filtered RNA-Seq reads using FastUniq v1.1^126^. The resultant reads were mapped to the coding genes using BWA-MEM v0.7.17^105^, and SAMtools v1.9^108^ was used to generate the alignment in BAM format. Using this alignment, BCFtools^127^ (v1.9) “mpileup” was used for variant calling in the coding genes, and for further filtering of false-positive variants based on the following parameters^128^—mapping quality ≥50, variant sites with quality ≥30, sequencing depth ≥30.

### Orthogroups identification

Representative species from all 15 monocot genus available in Ensembl plants release 47, and model organism *Arabidopsis thaliana* as an outgroup species were selected for orthogroups identification^129^. To construct the orthogroups, the protein sequences of *C. longa* obtained from TransDecoder and proteome files for other 16 selected species i.e., *Aegilops tauschii*, *Ananas comosus, Brachypodium distachyon*, *Dioscorea rotundata*, *Eragrostis tef*, *Hordeum vulgare*, *Leersia perrieri*, *Musa acuminata*, *Oryza sativa*, *Panicum hallii fil2*, *Saccharum spontaneum*, *Setaria italica*, *Sorghum bicolor*, *Triticum aestivum*, *Zea mays*, and *Arabidopsis thaliana* obtained from Ensembl release 47, were used. The longest isoforms for all proteins were extracted for all selected species to construct the orthogroups using OrthoFinder v2.3.9^40^.

### Construction of orthologous gene set and phylogenetic tree

Only those orthogroups that contained genes from all 17 species were extracted from all the identified orthogroups. The fuzzy one-to-one orthogroups containing genes from all 17 species were identified from these orthogroups, and extracted using KinFin v1.0^41^. For cases where the orthologous gene sets comprised of multiple genes for a species, the longest gene was extracted. The fuzzy one-to-one orthogroups were further aligned individually using MAFFT v7.467 for species phylogenetic tree construction^130^. BeforePhylo v0.9.0 (https://github.com/qiyunzhu/BeforePhylo) was used to trim the multiple sequence alignments to remove empty sites and to concatenate the multiple sequence alignments of all fuzzy one-to-one orthologous gene sets across 17 species. This concatenated alignment was used by the rapid hill climbing algorithm-based RAxML v8.2.12 for construction of maximum likelihood species phylogenetic tree with ‘PROTGAMMAAUTO’ amino acid substitution model using 100 bootstrap values^131^.

### Identification of genes with higher nucleotide divergence

Protein sequences of all the orthogroups across 17 species were aligned individually using MAFFT v7.467^130^, and individual maximum likelihood-based phylogenetic trees were built using these alignments by RAxML v8.2.12 (‘PROTGAMMAAUTO’ amino acid substitution model, 100 bootstrap values)^131^. The ‘adephylo’ package in R was used to calculate root-to-tip branch length distance values for each species^132^. *C. longa* genes that showed comparatively higher root-to-tip branch length with respect to the other selected species were extracted, and were considered as the genes with higher nucleotide divergence or higher rate of evolution.

### Identification of genes with unique substitution having functional impact

The unique substitutions in genes that have impact on protein function can identify species-specific amino acid substitutions and are considered as a site-specific evolutionary signature. However, the inclusion of phylogenetically distant species in this analysis may erroneously increase the number of uniquely substituted genes; therefore we restricted this analysis by only considering the monocot species (available on the Ensembl plant release 47) for reliable results. The amino acid positions that were identical across the other 16 species in the individual multiple sequence alignments of all orthogroups but different in *C. longa* were considered as the uniquely substituted amino acid positions.

For the identification of uniquely substituted sites, an in-house python script was used. Any gap and ten amino acid sites around any gap in the alignments were not considered in this analysis. The impact of these unique amino acid substitutions on protein function was identified using sorting intolerant from tolerant (SIFT), by utilizing UniProt as the reference database^133,134^.

### Identification of positively selected genes

The nucleotide sequences of all the orthogroups across 17 species were aligned using MAFFT v7.467^130^. ‘codeml’ from PAML package v4.9a that uses a branch-site model was used to identify positively selected genes using nucleotide alignments of all the orthologs in phylip format and the species phylogenetic tree generated in the previous steps^135^. Log-likelihood values were used to perform likelihood ratio tests and chi-square analysis-based p-values were calculated. The genes that qualified against the null model (fixed omega) (FDR-corrected *p*-values < 0.05) were identified as positively selected genes. All codon sites showing greater than 95% probability for foreground lineage based on Bayes Empirical Bayes (BEB) analysis were termed as positively selected sites.

### Genes with multiple signs of adaptive evolution (MSA)

Among the three signs of adaptive evolution—higher nucleotide divergence, unique substitution having functional impact and positive selection, the *C. longa* genes that showed at least two of these signs were termed as genes with multiple signs of adaptive evolution or MSA genes^136^. MSA (multiple signs of adaptive evolution) genes are obtained by taking the intersection of the genes showing different evolutionary signatures, and because of the presence of more than one evolutionary signature, these genes can be considered as the highly evolved genes. Thus, these genes are useful to decipher and to strongly support the mechanisms or pathways responsible for adaptive evolution of the species.

### Functional annotation

KAAS genome annotation server v2.1 was used to assign KEGG Orthology (KO) identifiers and KEGG pathways to the genes^137^. eggNOG-mapper v2 was used for functional annotation of genes using precomputed orthologous groups from eggNOG clusters^138^. WebGeStalt web server was used for GO enrichment analysis, and only the GO categories showing *p*-values < 0.05 in over-representation enrichment analysis were considered further^139^. The assignment of genes into functional categories was manually curated.

### Curcuminoid biosynthesis pathway

Coding sequences of four key genes involved in curcuminoid biosynthesis pathway, namely curcumin synthase 1 (*CURS1*, NCBI accession number BAH56226), curcumin synthase 2 (*CURS2*, NCBI accession number AB506762), curcumin synthase 3 (*CURS3*, NCBI accession number AB506763), and diketide-CoA synthase (*DCS*, NCBI accession number BAH56225) were retrieved^140^. The sequences of these four genes were mapped to the gene set derived from de novo transcriptome assembly generated in this study, and the gene set derived from MAKER annotation pipeline using BLASTN^117^ with query coverage ≥50% and e-value 10^−9^. These sequences were also aligned to de novo genome assembly of *C. longa* constructed in this study, using Exonerate v2.2.0 (https://github.com/nathanweeks/exonerate) with 95% of maximal alignment score and 95% quality threshold, and the best hits were selected to construct the gene structures.

Further, the ten enzymes involved in curcuminoid biosynthesis pathway (Fig. 3a) were searched, and identified in the proteome sequences of *C. longa* using CAPS_protocol^81^. EC numbers or NCBI accession numbers of these ten enzymes (Supplementary Table 22) were used for homolog identification for each enzyme in UniProt database^134^, and the top hits that were found in UniProt database were retained. These homolog sequences were then aligned using Clustal Omega v1.2.4 (https://www.ebi.ac.uk/Tools/msa/clustalo/) (default parameters). In order to identify the true homologs, the functionally important residues (FIR)-binding site and active site amino acid residues for each enzyme (Supplementary Table 23) were detected from UniProt^134^ database, and sequences that did not contain those residues were removed from the alignments. These filtered alignments were queried against the proteome sequences of *C. longa* using PSI-BLAST^141^ with e-value of 10^−5^, inclusion threshold of 10^−5^, query coverage ≥70%, sequence identity ≥40%, and 2 iterations, as used in CAPS_protocol^81^. The PSI-BLAST hits were again searched for the presence of FIRs, and the best identical hits were retained.

### Evolution and phylogenetic analysis of curcuminoid biosynthesis pathway in *C. longa*

In order to elucidate the origin of the candidate enzymes involved in curcuminoid biosynthesis pathway, the ten genes identified in the previous step were used for phylogenetic analysis of these enzymes. The amino acid sequences of the identified genes were mapped against UniRef30 database^120^ using HHblits^142^ web server (default parameters). The top 20 hits were searched to extract one gene for each unique genus, and the target sequences were mapped for sequence domains using Pfam-A (v32.0) database, and only those sequences with the identified domains for each enzyme were selected as candidate homologs of the corresponding enzymes. The selected homologs were aligned using MAFFT v7.467, the empty sites were removed from the multiple sequence alignments using BeforePhylo v0.9.0, and the filtered alignments were used for construction of maximum likelihood-based gene phylogenetic tree for individual genes using RAxML v8.2.12 with bootstrap values of 1000 and ‘PROTGAMMAAUTO’ amino acid substitution model.

CAFÉ v4.2.1^84^ was used to analyse the evolution of the gene families that included the genes involved in curcuminoid biosynthesis pathway. The protein sequences of the selected 17 plant species (including *Arabidopsis thaliana* as an outgroup) were used for all-versus-all BLASTP homology search, and subsequent clustering using MCL^143^ v14.137. After clustering, the gene families that contained ≥100 gene copies for at least one species were removed. The filtered gene families and the ultrametric species phylogenetic tree were used for gene family expansion and contraction analysis using two-lambda (λ) model. In this two-lambda (λ) model, the clade formed by *C. longa* and *Musa acuminata* was assigned separate λ-value compared to the rest of the species (Supplementary Fig. 4).

### Statistics and reproducibility

Computational data analyses were performed using Linux, Perl, and Python custom scripts. Statistical tests (chi-square, Bayes Empirical Bayes) used in positive selection analysis were performed using PAML v4.9a^135^. Statistical significance levels are mentioned as *p* < 0.05. Statistically significant GO enriched categories were analyzed using WebGeStalt web server^139^. Branch length distance values were calculated for higher nucleotide divergence analysis using ‘adephylo’^132^ package in R v3.6.0. For DNA–RNA extraction and sequencing, a single plant individual (*n* = 1) was used.

### Reporting summary

Further information on research design is available in the Nature Research Reporting Summary linked to this article.

## Supplementary information

Supplementary Information

Description of Additional Supplementary Files

Supplementary Data 1

Reporting Summary

## Data Availability

The raw genome and transcriptome reads of *C. longa* have been deposited in National Center for Biotechnology Information (NCBI) Sequence Read Archive (SRA) under BioProject accession—PRJNA660606, BioSample accession—SAMN15954062, SRA accessions—SRR12560783, SRR12560784, SRR12560785, SRR15204660, SRR15204661. Detailed information related to the MSA genes of *C. longa* have been provided in Supplementary Data 1.
