# Supplementary material for: Genome sequencing of turmeric provides evolutionary insights into its medicinal properties
Source: Commun Biol. 2021 Oct 15;4:1193. doi: 10.1038/s42003-021-02720-y (PMC8521574; doi:10.1038/s42003-021-02720-y)
Supplement: Supplementary file 1 — Supplementary Information [file 42003_2021_2720_MOESM1_ESM.pdf]

SUPPLEMENTARY INFORMATION

Title: Genome sequencing of turmeric provides evolutionary insights into its medicinal properties

Authors: Abhisek Chakraborty[1], Shruti Mahajan[1], Shubham K. Jaiswal[1], Vineet K. Sharma[1*]

Affiliation:

[1]MetaBioSys Group, Department of Biological Sciences, Indian Institute of Science Education and Research Bhopal, Bhopal, India

*Corresponding Author email:

Vineet K. Sharma - vineetks@iiserb.ac.in

Email addresses of authors:

Abhisek Chakraborty - abhisek18@iiserb.ac.in, Shruti Mahajan - shruti17@iiserb.ac.in, Shubham K. Jaiswal - shubhamj@iiserb.ac.in, Vineet K. Sharma - vineetks@iiserb.ac.in

SUPPLEMENTARY TABLES

Supplementary Table 1. Summary of the 10x Genomics linked read sequence data generated for *Curcuma longa* genome

Average Read Length	Number of Reads	Total Data	Sequencing data coverage
150 bp	631.11 million	94.8 Gb	~82.4X

The sequencing coverage was calculated with respect to the estimated genome size of 1.15 Gb.

Supplementary Table 2. Summary of RNA-Seq data used for *C. longa* transcriptome analysis

SRA accession No.	Average Read Length R1 (bp)	Average Read Length R2 (bp)	Total Number of Read pairs	Total Number of Bases (bp)	Reference
SRR12560783 and SRR15204660	147.4	147.4	105,525,957(x2)	32,402,194,954	This study
RNA-Seq data used from other studies as empirical evidence in MAKER pipeline					
SRX969036	100	100	33,384,838(x2)	6,676,967,600	[1]
SRX146854	72	72	20,519,880(x2)	2,954,862,720	[2]
SRX146981	73	73	30,342,598(x2)	4,430,019,308	[2]
SRX146982	100	100	37,193,403(x2)	7,438,680,600	[2]
SRX1829461	76	76	25,797,778(x2)	3,921,262,256	[3]
SRX2033300	76	76	30,091,732(x2)	4,573,943,264	[4]
ERX2099818 and ERX2099819	90	90	33,743,100(x2)	6,073,758,000	[5]

In addition to our data, RNA-Seq data from previously reported studies were used as empirical evidence in MAKER pipeline, which resulted in the total transcriptome data of 68.5 Gb[1–5].

Supplementary Table 3. Summary statistics of final *de novo* draft genome assembly of *C. longa*

Parameter	Value
Number of contigs (>= 1000 bp)	30,301
Number of contigs (>= 3000 bp)	22,470
Number of contigs (>= 5000 bp)	19,403
Number of contigs (>= 10000 bp)	15,525
Number of contigs (>= 25000 bp)	10,001
Number of contigs (>= 50000 bp)	5,836
Total length (>= 1000 bp)	1,038,849,290
Total length (>= 3000 bp)	1,024,351,399
Total length (>= 5000 bp)	1,012,428,174
Total length (>= 10000 bp)	984,434,743
Total length (>= 25000 bp)	893,164,070
Total length (>= 50000 bp)	743,020,613
Largest contig (bp)	1,867,898
N50 (>= 1000 bp)	98,563
N50 (>= 3000 bp)	100,562
GC % (>= 3000 bp)	38.75
L50 (>= 1000 bp)	2,592
L50 (>= 3000 bp)	2,519
Number of N's per 100 kbp (>= 3000 bp)	91.76

Supplementary Table 4. BUSCO statistics of *C. longa* genome

Parameters	Supernova v2.1.1 assembled raw genome	Flye v2.4.2 assembled raw genome	Final *C. longa* draft genome
Complete BUSCOs (C)	1,226 (75.9%)	1,154 (71.5%)	1,490 (92.4%)
Fragmented BUSCOs (F)	169 (10.5%)	169 (10.5%)	36 (2.2%)
Missing BUSCOs (M)	219 (13.6%)	291 (18%)	88 (5.4%)
Total BUSCO groups searched	1,614	1,614	1,614

BUSCO analysis was performed using the reference BUSCO database embryophyta_odb10

Supplementary Table 5. LTR assembly index (LAI) values of *C. longa* genome assembly

Assembly cut-off	Genome size (bases)	LAI score	Category
>= 3 Kb	1,024,351,399	8.01	Draft
>= 5 Kb	1,012,428,174	8.18	Draft
>= 10 Kb	984,434,743	8.61	Draft
>= 35 Kb	832,217,381	10.26	Reference

Note: The LAI score-based category was determined based on the classification given by Ou et al. (2018)[6]

Supplementary Table 6. Summary statistics of *de novo* transcriptome assembly of *C. longa*

Statistics based on all transcript contigs		
	de novo **transcriptome assembly (≥500 bp) using data from this study**	***de novo*** **transcriptome assembly using data from this study and previous reports**
Contig N50	1,086	1,531
Median contig length	798	505
Average contig	1,019.38	899.74

Total assembled bases	86,158,097	383,724,313
**Statistics based on only longest isoform per gene**		
Contig N50	1,008	940
Median contig length	762	338
Average contig	961.43	621.99
Total assembled bases	35,101,755	123,593,801
**Counts of genes and transcripts**		
Total trinity 'genes'	36,510	198,706
Total trinity transcripts	84,520	426,484
GC (%)	45.45	42.69

Supplementary Table 7. Summary statistics of repetitive sequences in *C. longa* genome identified by RepeatMasker

Total length (>= 1,000 bp):	1,038,849,290 bp				
GC (%)	38.79%				
Bases masked:	666,515,997 bp (64.16%)				
			Number of Elements	Length occupied (bp)	Percentage of Sequence
Retroelements			283,516	296,063,884 bp	28.50%
	SINEs		0	0 bp	0.00%
	Penelope		0	0 bp	0.00%
	LINEs		20,928	11,698,986 bp	1.13%
		CRE/SLACS	0	0 bp	0.00%

		L2/CR1/Rex	0	0 bp	0.00%
		R1/LOA/Jockey	204	109,609 bp	0.01 %
		R2/R4/NeSL	0	0 bp	0.00%
		RTE/Bov-B	15,129	8,569,893 bp	0.82 %
		L1/CIN4	5,595	3,019,484 bp	0.29 %
	LTR elements:		262,588	284,364,898 bp	27.37 %
		BEL/Pao	0	0 bp	0.00%
		Ty1/Copia	156,608	178,624,852 bp	17.19 %
		Gypsy/DIRS1	96,511	97,823,063 bp	9.42 %
		Retroviral	273	205,016 bp	0.02 %
DNA transposons			34,533	23,473,676 bp	2.26 %
	hobo-Activator		10,926	6,114,321 bp	0.59 %
	Tc1-IS630-Pogo		0	0 bp	0.00%
	En-Spm		0	0 bp	0.00%
	MuDR-IS905		0	0 bp	0.00%
	PiggyBac		0	0 bp	0.00%
	Tourist/Harbinger		2,839	1,414,557 bp	0.14 %
	Other (Mirage, P-element, Transib)		0	0 bp	0.00%
Rolling-circles			15,232	4,921,881 bp	0.47 %

Unclassified:			897,969	328,428,756 bp	31.61 %
Total interspersed repeats:				647,966,316 bp	62.37 %
Small RNA:			7,925	4,718,997 bp	0.45 %
Satellites:			1,444	164,666 bp	0.02%
Simple repeats:			147,240	7,269,924 bp	0.70 %
Low complexity:			27,915	1,474,213 bp	0.14 %

Note: The information provided in the above table is as per the default output of RepeatMasker program.

Supplementary Table 8. Distribution of genes of *C. longa* with higher rate of evolution in different KEGG pathways

**KEGG pathway**	**Number of genes**
Plant hormone signal transduction	2
Photosynthesis	2
PI3K-Akt signaling pathway	2
Endocytosis	2
Phenylpropanoid biosynthesis	1
Glutathione metabolism	1
MAPK signaling pathway – plant	1
Fructose and mannose metabolism	1
Methane metabolism	1
Glycerolipid metabolism	1
Glycerophospholipid metabolism	1

Sphingolipid metabolism	1
Phenylalanine, tyrosine and tryptophan biosynthesis	1
Selenocompound metabolism	1
Other glycan degradation	1
Metabolism of xenobiotics by cytochrome P450	1
Ribosome	1
Aminoacyl-tRNA biosynthesis	1
RNA transport	1
Protein processing in endoplasmic reticulum	1
SNARE interactions in vesicular transport	1
Ubiquitin mediated proteolysis	1
Proteasome	1
Phagosome	1
Lysosome	1
Cell cycle	1
NOD-like receptor signaling pathway	1
Circadian rhythm - plant	1
Longevity regulating pathway	1
Mineral absorption	1

Supplementary Table 9. Distribution of positively selected genes of *C. longa* in different KEGG pathways (Pathways with >1 gene are mentioned)

KEGG pathway	Number of genes

Purine metabolism	6
Pyruvate metabolism	5
Ribosome	5
Endocytosis	5
Cellular senescence	5
Starch and sucrose metabolism	4
Porphyrin and chlorophyll metabolism	4
Spliceosome	4
Glycolysis / Gluconeogenesis	3
Pentose and glucuronate interconversions	3
alpha-Linolenic acid metabolism	3
Tyrosine metabolism	3
Phenylalanine metabolism	3
Phenylalanine, tyrosine and tryptophan biosynthesis	3
RNA polymerase	3
Protein processing in endoplasmic reticulum	3
RNA degradation	3
Ubiquitin mediated proteolysis	3
MAPK signaling pathway – plant	3
Calcium signaling pathway	3
Phosphatidylinositol signaling system	3
AMPK signaling pathway	3
Plant hormone signal transduction	3

Plant-pathogen interaction	3
Tight junction	3
Citrate cycle (TCA cycle)	2
Pentose phosphate pathway	2
Amino sugar and nucleotide sugar metabolism	2
Oxidative phosphorylation	2
Photosynthesis - antenna proteins	2
Methane metabolism	2
Fatty acid elongation	2
Fatty acid degradation	2
Glycerolipid metabolism	2
Glycerophospholipid metabolism	2
Biosynthesis of unsaturated fatty acids	2
Pyrimidine metabolism	2
Cysteine and methionine metabolism	2
Tryptophan metabolism	2
Glutathione metabolism	2
Thiamine metabolism	2
Terpenoid backbone biosynthesis	2
Phenylpropanoid biosynthesis	2
Basal transcription factors	2
RNA transport	2
Proteasome	2

Mismatch repair	2
MAPK signaling pathway	2
cAMP signaling pathway	2
cGMP-PKG signaling pathway	2
PI3K-Akt signaling pathway	2
Phagosome	2
Peroxisome	2
Cell cycle	2
NOD-like receptor signaling pathway	2

Supplementary Table 10. The distribution of genes of *C. longa* with positively selected codon sites in different KEGG pathways (Pathways with ≥5 genes are mentioned)

**KEGG pathway**	**Number of genes**
Ribosome	10
Purine metabolism	8
Ubiquitin mediated proteolysis	8
Pyruvate metabolism	7
Protein processing in endoplasmic reticulum	7
Cell cycle	7
Starch and sucrose metabolism	6
RNA transport	6
MAPK signaling pathway – plant	6
Pentose and glucuronate interconversions	5
Oxidative phosphorylation	5

alpha-Linolenic acid metabolism	5
Porphyrin and chlorophyll metabolism	5
Spliceosome	5
PI3K-Akt signaling pathway	5
Endocytosis	5
Cellular senescence	5

Supplementary Table 11. The distribution of genes of *C. longa* having unique substitution with functional impact in different KEGG pathways (Pathways with ≥10 genes are mentioned)

**KEGG pathway**	**Number of genes**
Spliceosome	27
RNA transport	26
Protein processing in endoplasmic reticulum	26
Ribosome	25
Cell cycle	24
Starch and sucrose metabolism	22
Ubiquitin mediated proteolysis	22
Glycolysis / Gluconeogenesis	21
Purine metabolism	20
Cysteine and methionine metabolism	20
Ribosome biogenesis in eukaryotes	20
Endocytosis	19
Pyruvate metabolism	18
Glycerolipid metabolism	18

Plant-pathogen interaction	18
Aminoacyl-tRNA biosynthesis	17
mRNA surveillance pathway	17
RNA degradation	17
AMPK signaling pathway	16
Plant hormone signal transduction	16
Amino sugar and nucleotide sugar metabolism	15
Carbon fixation in photosynthetic organisms	14
Phenylalanine, tyrosine and tryptophan biosynthesis	14
Peroxisome	14
Glutathione metabolism	13
MAPK signaling pathway – plant	13
Oxidative phosphorylation	12
Glycerophospholipid metabolism	12
Terpenoid backbone biosynthesis	12
Lysosome	12
Cellular senescence	12
Glyoxylate and dicarboxylate metabolism	11
Pyrimidine metabolism	11
Alanine, aspartate and glutamate metabolism	11
Glycine, serine and threonine metabolism	11
DNA replication	11
Citrate cycle (TCA cycle)	10

Methane metabolism	10
Fatty acid biosynthesis	10
Valine, leucine and isoleucine degradation	10
Arginine and proline metabolism	10
Proteasome	10
PI3K-Akt signaling pathway	10

Supplementary Table 12. The distribution of MSA genes of *C. longa* in different KEGG pathways (Pathways with more than one gene are mentioned)

**KEGG pathway**	**Number of genes**
Plant hormone signal transduction	4
Pyruvate metabolism	4
Endocytosis	4
Glycolysis / Gluconeogenesis	3
Starch and sucrose metabolism	3
Methane metabolism	3
Purine metabolism	3
Phenylalanine, tyrosine and tryptophan biosynthesis	3
Ubiquitin mediated proteolysis	3
MAPK signaling pathway – plant	3
Cell cycle	3
Cellular senescence	3
Pentose phosphate pathway	2

Fructose and mannose metabolism	2
Glycerolipid metabolism	2
alpha-Linolenic acid metabolism	2
Tyrosine metabolism	2
Phenylalanine metabolism	2
Tryptophan metabolism	2
Selenocompound metabolism	2
Glutathione metabolism	2
Thiamine metabolism	2
Porphyrin and chlorophyll metabolism	2
Terpenoid backbone biosynthesis	2
Phenylpropanoid biosynthesis	2
RNA polymerase	2
Spliceosome	2
Aminoacyl-tRNA biosynthesis	2
RNA transport	2
RNA degradation	2
Calcium signaling pathway	2
cGMP-PKG signaling pathway	2
PI3K-Akt signaling pathway	2
AMPK signaling pathway	2
NOD-like receptor signaling pathway	2
Plant-pathogen interaction	2

Supplementary Table 13. The distribution of genes of *C. longa* with higher rate of evolution in different COG categories (obtained from eggNOG-mapper v2)

COG category	Number of genes
Function unknown	15
Post-translational modification, protein turnover, and chaperones	7
Signal transduction mechanisms	6
Transcription	6
Carbohydrate transport and metabolism	6
Translation, ribosomal structure and biogenesis	5
Cell wall/membrane/envelope biogenesis	4
Intracellular trafficking, secretion, and vesicular transport	4
RNA processing and modification	4
Cell cycle control, cell division, chromosome partitioning	2
Amino acid transport and metabolism	2
Lipid transport and metabolism	2

Note: COG categories with >1 gene are mentioned above

Supplementary Table 14. The distribution of positively selected genes of *C. longa* in different COG categories (obtained from eggNOG-mapper v2)

COG category	Number of genes
Function unknown	72
Post-translational modification, protein turnover, and chaperones	36

Signal transduction mechanisms	31
Carbohydrate transport and metabolism	27
Transcription	24
Inorganic ion transport and metabolism	17
Translation, ribosomal structure and biogenesis	14
Energy production and conversion	14
Secondary metabolites biosynthesis, transport, and catabolism	11
Nucleotide transport and metabolism	10
Intracellular trafficking, secretion, and vesicular transport	10
Cell cycle control, cell division, chromosome partitioning	9
Lipid transport and metabolism	9
Amino acid transport and metabolism	8
Replication, recombination and repair	7
RNA processing and modification	7
Cytoskeleton	6
Coenzyme transport and metabolism	6
Chromatin structure and dynamics	4
Defense mechanisms	3
Cell wall/membrane/envelope biogenesis	3
Nuclear structure	2

Note: COG categories with >1 gene are mentioned above

Supplementary Table 15. The distribution of genes of *C. longa* with positively selected codon sites in different COG categories (obtained from eggNOG-mapper v2)

COG category	Number of genes
Function unknown	147
Post-translational modification, protein turnover, and chaperones	63
Signal transduction mechanisms	58
Transcription	44
Carbohydrate transport and metabolism	44
Inorganic ion transport and metabolism	27
Translation, ribosomal structure and biogenesis	26
Energy production and conversion	23
Secondary metabolites biosynthesis, transport, and catabolism	20
Intracellular trafficking, secretion, and vesicular transport	18
Replication, recombination and repair	18
Cell cycle control, cell division, chromosome partitioning	16
Lipid transport and metabolism	16
Amino acid transport and metabolism	15
RNA processing and modification	14
Nucleotide transport and metabolism	12
Cytoskeleton	11
Cell wall/membrane/envelope biogenesis	9

Coenzyme transport and metabolism	8
Chromatin structure and dynamics	7
Defense mechanisms	4
Nuclear structure	2

Note: COG categories with >1 gene are mentioned above

Supplementary Table 16. The distribution of genes of *C. longa* showing unique substitution with functional impact in different COG categories (obtained from eggNOG-mapper v2)

COG category	Number of genes
Function unknown	571
Signal transduction mechanisms	274
Post-translational modification, protein turnover, and chaperones	228
Carbohydrate transport and metabolism	190
Transcription	163
Translation, ribosomal structure and biogenesis	134
Intracellular trafficking, secretion, and vesicular transport	129
RNA processing and modification	101
Lipid transport and metabolism	98
Amino acid transport and metabolism	95
Secondary metabolites biosynthesis, transport, and catabolism	89
Inorganic ion transport and metabolism	80
Energy production and conversion	75

Cell cycle control, cell division, chromosome partitioning	67
Replication, recombination and repair	60
Cytoskeleton	51
Cell wall/membrane/envelope biogenesis	43
Nucleotide transport and metabolism	38
Chromatin structure and dynamics	33
Coenzyme transport and metabolism	33
Defense mechanisms	20
Nuclear structure	10
Extracellular structures	9

Note: COG categories with >1 gene are mentioned above

Supplementary Table 17. The distribution of MSA genes of *C. longa* in different COG categories (obtained from eggNOG-mapper v2)

COG category	Number of genes
Function unknown	45
Post-translational modification, protein turnover, and chaperones	21
Signal transduction mechanisms	21
Carbohydrate transport and metabolism	20
Transcription	15
Inorganic ion transport and metabolism	11
Cell cycle control, cell division, chromosome partitioning	9

Translation, ribosomal structure and biogenesis	9
Secondary metabolites biosynthesis, transport, and catabolism	7
Lipid transport and metabolism	6
Amino acid transport and metabolism	6
Energy production and conversion	6
Intracellular trafficking, secretion, and vesicular transport	6
Cytoskeleton	5
Coenzyme transport and metabolism	4
Nucleotide transport and metabolism	4
Replication, recombination and repair	4
RNA processing and modification	3

Note: COG categories with >1 gene are mentioned above

Supplementary Table 18. The biological process GO categories that were enriched in *C. longa* MSA genes (Only statistically significant GO terms with p-values<0.05 are mentioned)

GO term ID	Description	p-value
GO:0022613	ribonucleoprotein complex biogenesis	0.002
GO:0006753	nucleoside phosphate metabolic process	0.006
GO:0090407	organophosphate biosynthetic process	0.008
GO:0034660	ncRNA metabolic process	0.01

GO:0071826	ribonucleoprotein complex subunit organization	0.02
GO:0071669	plant-type cell wall organization or biogenesis	0.04

Note: GO analysis was performed using non-redundant Biological Process database in WebGeStalt

Supplementary Table 19. The cellular component GO categories that were enriched in *C. longa* MSA genes (Only statistically significant GO terms with p-values<0.05 are mentioned)

GO term ID	Description	p-value
GO:0030684	Preribosome	0.037

Note: GO analysis was performed using non-redundant Cellular component database in WebGeStalt

Supplementary Table 20. The molecular function GO categories that were enriched in *C. longa* MSA genes (Only statistically significant GO terms with p-values<0.05 are mentioned)

GO term ID	Description	p-value
GO:0032182	ubiquitin-like protein binding	0.013
GO:0042393	histone binding	0.037

Note: GO analysis was performed using non-redundant Molecular Function database in WebGeStalt

Supplementary Table 21. Function of MSA genes identified in *C. longa* in secondary metabolite biosynthesis

Gene name	Gene description	Activity	Medicinal properties*
CHI	Chalcone isomerase	Flavonoids (Anthocyanin) biosynthesis	AO, AD, AI, anti-obesity, etc.
ADT	Arogenate dehydratase	Lignin, anthocyanin biosynthesis	AO, AC, AD, AI, anti-obesity, etc.

GST	Glutathione S-Transferase	Glucosinolate biosynthesis	AC, AM
SK	Shikimate kinase	Phenolic compounds (flavonoid, isoflavonoid, anthraquinone, chalcone etc.) biosynthesis	AI, AO, AC, AM, anti-proliferative, etc.
MK	Mevalonate kinase	Isoprenoid, sterol biosynthesis	AC, AM, AO, AI, immunomodulatory, etc.
PAL	Phenylalanine ammonia-lyase	Phenylpropanoid, curcuminoid, alkaloid biosynthesis	AC, AI, AO, AC, anti-allergic, etc.
DWF4	Dwarf 4	Brassinosteroid biosynthesis	AI, AM, anti-angiogenic, etc.
AT1G04430	S-adenosyl-L-methionine-dependent methyltransferases superfamily protein	Lignin, phenylpropanoid, flavonoids, anthocyanin, terpenoid, alkaloid production	AD, AO, AM, AI, AC, etc.
CYP706A1	Cytochrome P450, Family 706, Subfamily A, Polypeptide 1	flavonoid, alkaloid, terpenoids, furanocoumarins, glucosinolates, allelochemicals biosynthesis	AO, AM, AI, AC, etc.

[*]Abbreviations: AC = anti-cancer, AO = anti-oxidant, AM = anti-microbial, AD = anti-diabetic, AI = anti-inflammatory.

Supplementary Table 22. Details of enzymes involved in curcuminoid biosynthesis pathway

Name of the enzyme	Enzymatic step	EC number	Number of *C. longa* genes present in the corresponding gene families	Expansion/contraction of the gene family
Phenylalanine ammonia lyase (*PAL*)	Phenylalanine -> cinnamic acid	EC:4.3.1.24	9	Expanded (+1)
Cinnamate-4-hydroxylase (*C4H*)	Cinnamic acid -> p-coumaric acid	EC:1.14.14.91	8	Expanded (+2)
4-coumarate-CoA ligase (*4CL*)	p-coumaric acid -> p-coumaroyl-CoA	EC:6.2.1.12	35	Expanded (+10)
Hydroxycinnamoyl transferase (*HCT*)	p-coumaroyl-CoA -> feruloyl-CoA	EC:2.3.1.133	11	Contracted (-3)
Cinnamate-3-hydroxylase (*C3H*)	p-coumaroyl-CoA -> feruloyl-CoA	EC:1.14.14.96	49	Expanded (+5)
O-methyltransferase (*OMT*)	p-coumaroyl-CoA -> feruloyl-CoA	EC:2.1.1.104	6	Contracted (-2)
Diketide-CoA synthase (*DCS*)	p-coumaroyl-CoA -> p-coumaroyl-diketide-CoA<br>feruloyl-CoA -> feruloyl-diketide-CoA	EC:2.3.1.218	27	Expanded (+6)
Curcumin synthase 1 (*CURS1*)	p-coumaroyl-diketide-CoA -> demethoxycurcumin<br>feruloyl-diketide-CoA -> demethoxycurcumin and curcumin	EC:2.3.1.217	27	Expanded (+6)

Curcumin synthase 2 (*CURS2*)	p-coumaroyl-diketide-CoA -> demethoxycurcumin<br>feruloyl-diketide-CoA -> demethoxycurcumin and curcumin	EC:2.3.1.219	27	Expanded (+6)
Curcumin synthase 3 (*CURS3*)	p-coumaroyl-diketide-CoA -> bisdemethoxycurcumin and demethoxycurcumin<br>feruloyl-diketide-CoA -> demethoxycurcumin and curcumin	EC:2.3.1.219	27	Expanded (+6)

Note: *DCS*, *CURS1*, *CURS2* and *CURS3* genes were present in the same gene family obtained from CAFÉ analysis

Supplementary Table 23. Functionally Important Residues (FIR) information for enzymes involved in curcuminoid biosynthesis pathway

Enzyme name	Binding site	Active site	Reference Swiss-Prot sequence
Phenylalanine ammonia lyase (*PAL*)	Asparagine (N269), Glutamine (Q357), Arginine (R363), Asparagine (N393), Asparagine (N496)	Tyrosine (Y117)	P35510_ARATH
Cinnamate-4-hydroxylase (*C4H*)	Alanine (A306)	_	P92994_ARATH
4-coumarate-CoA ligase (*4CL*)	Histidine (H261), Threonine (T361), Aspartate (D442),	_	Q84P24_ARATH

	Arginine (R457), Lysine (K548)		
Hydroxycinnamoyl transferase (*HCT*)	–	Histidine (H153), Aspartate (D380)	Q9FI78_ARATH
Cinnamate-3-hydroxylase (*C3H*)	–	–	–
O-methyltransferase (*OMT*)	Lysine (K33), Threonine (T75), Glutamate (E97), Serine (S105), Aspartate (D123), Alanine (A152), Aspartate (D175), Aspartate (D177), Asparagine (N206)	–	O49499_ARATH
Diketide-CoA synthase (*DCS*)	Threonine (T200), Alanine (A311)	Cysteine (C167)	Q2R3A1_ORYSJ
Curcumin synthase 1 (*CURS1*)	–	Cysteine (C164)	C0SVZ6_CURLO
Curcumin synthase 2 (*CURS2*)	–	Cysteine (C166)	C6L7V8_CURLO
Curcumin synthase 3 (*CURS3*)	–	Cysteine (C164)	C6L7V9_CURLO

SUPPLEMENTARY FIGURES

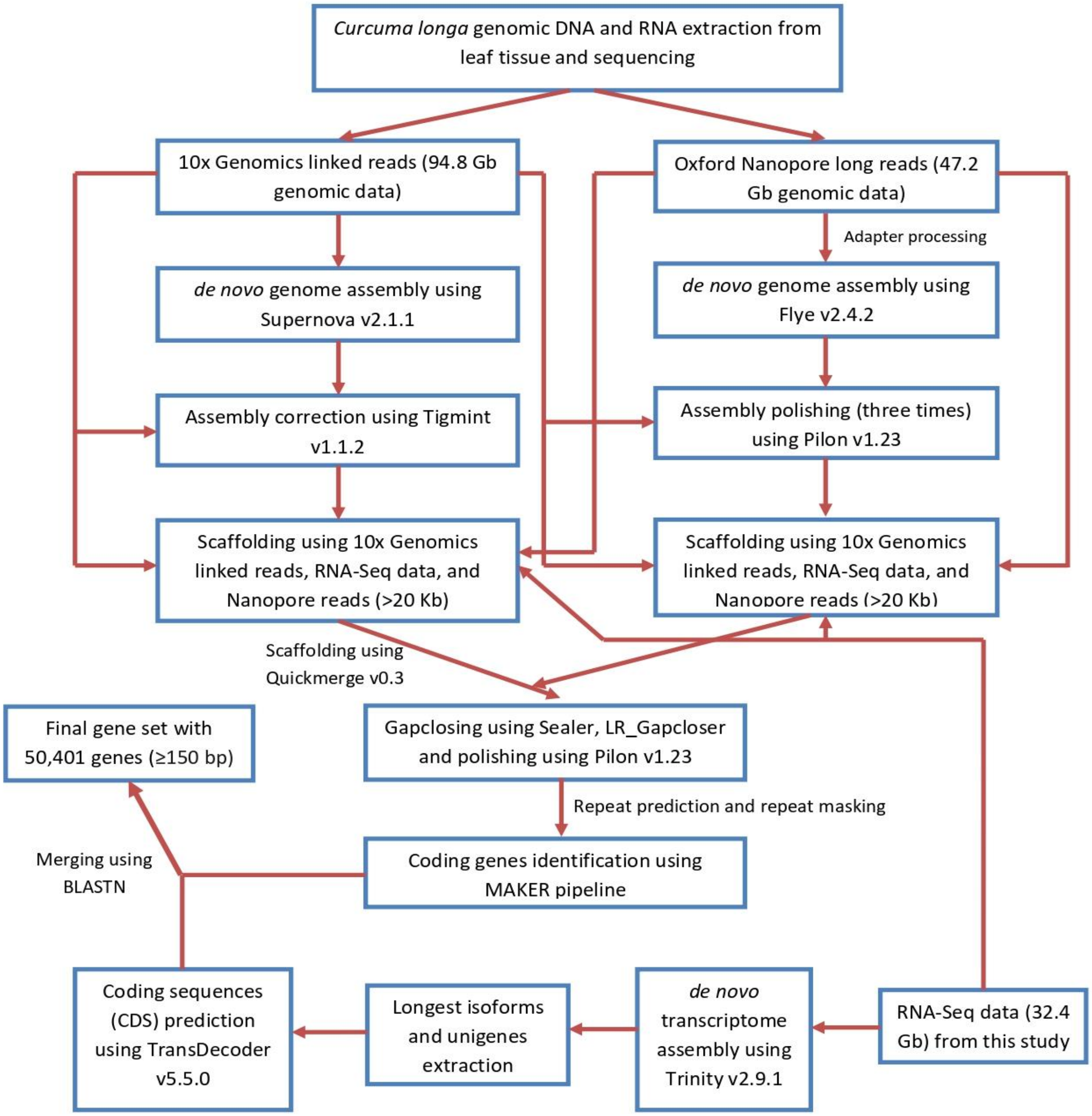

Supplementary Figure 1. The complete workflow of the genome and transcriptome analysis of *C. longa*

a.

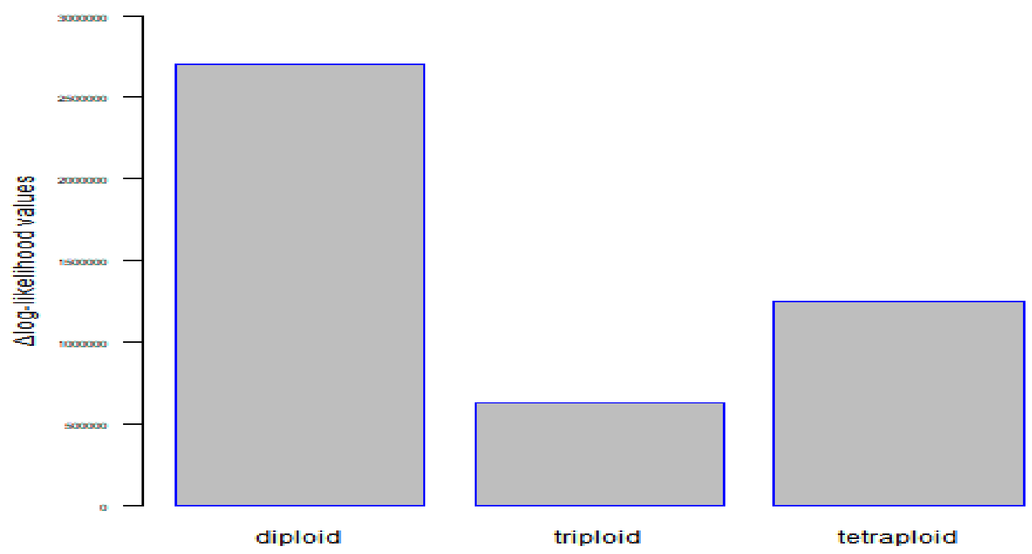

b.

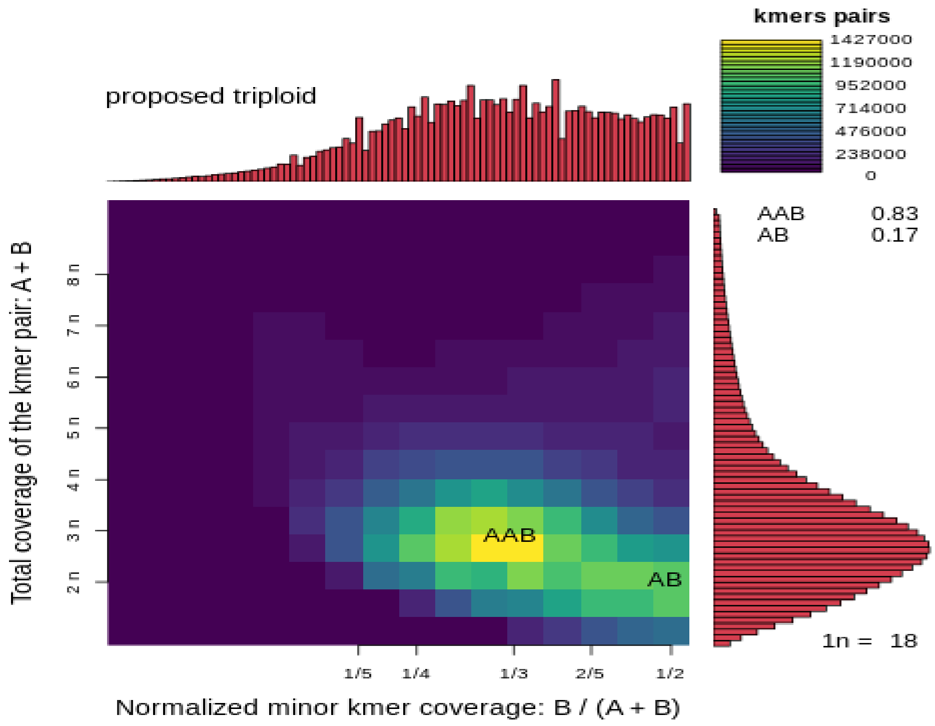

Supplementary Figure 2. Ploidy level estimation for *C. longa* genome. **a.** Δlog-likelihood values (after denoising) for diploid, triploid, tetraploid fixed models obtained using nQuire (Note: minimum mapping

quality was set to 30 in nQuire analysis), **b.** Smudgeplot for the haplotype structure of *C. longa* genome obtained from heterozygous k-mer pairs.

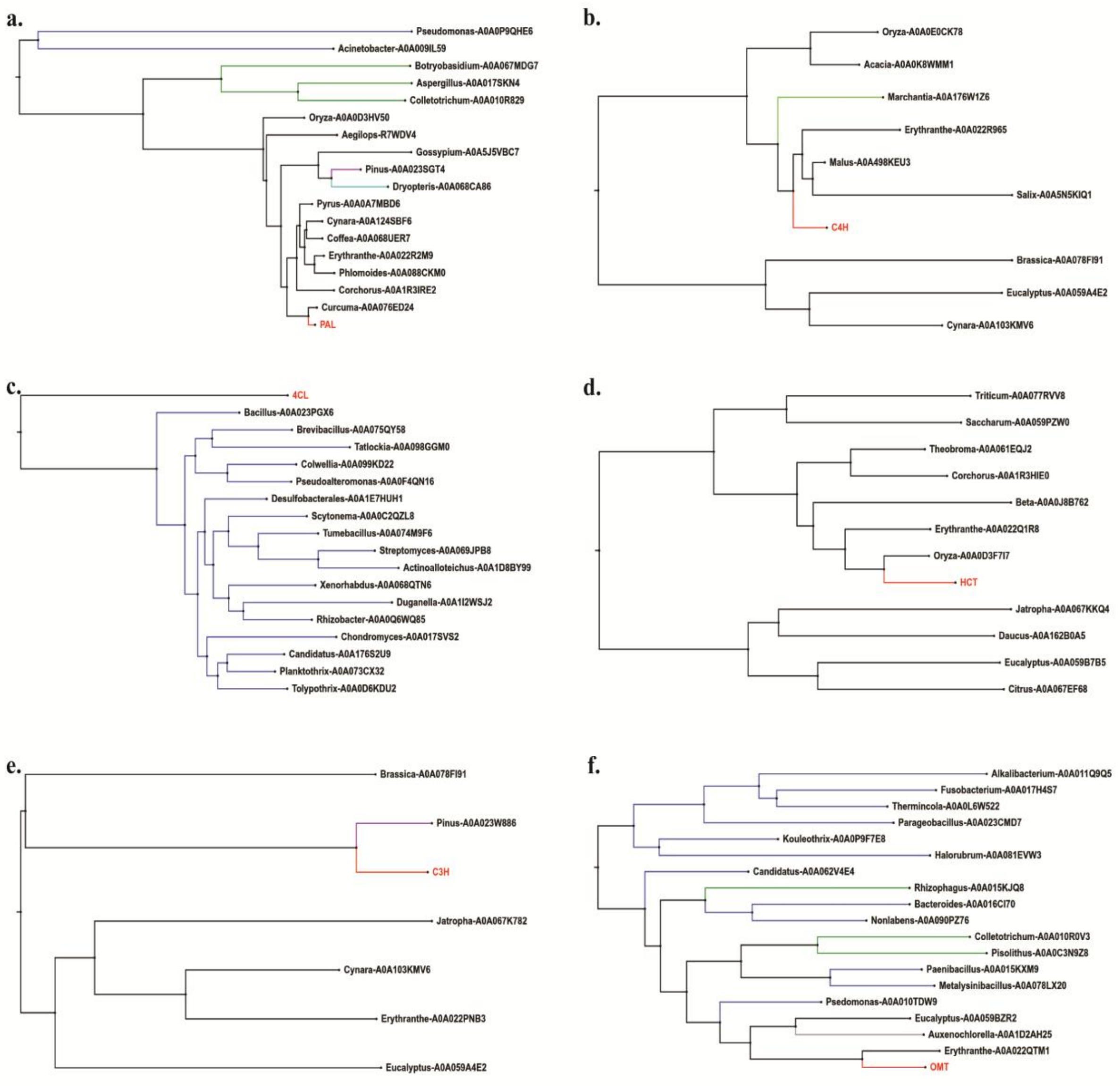

Supplementary Figure 3. Phylogenetic relationship of the candidate enzymes of upper curcuminoid biosynthesis pathway in *C. longa* with their distant orthologous genes. Blue coloured line denotes Bacterial orthologs, Grey coloured line denotes Algal ortholog, Dark green coloured line denotes Fungal orthologs, Light green coloured line denotes ortholog from Bryophyte, Sky blue coloured line denotes

ortholog from Pteridophyte, Black coloured line denotes Angiosperm orthologs, Red coloured line denotes the genes of interest in *C. longa*, and Violet coloured line denotes orthologs from Gymnosperm. **a.** *PAL* gene, **b.** *C4H* gene, **c.** *4CL* gene, **d.** *HCT* gene, **e.** *C3H* gene, **f.** *OMT* gene.

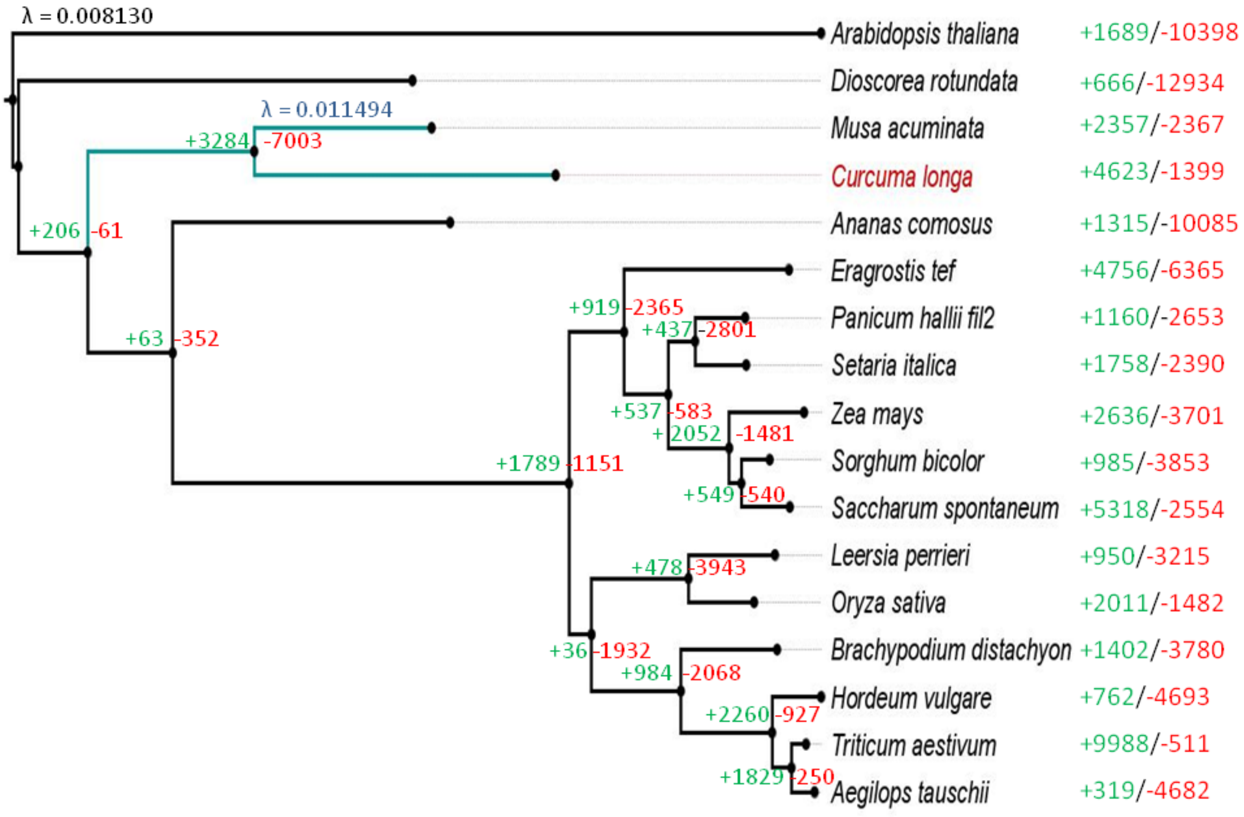

Supplementary Figure 4. Expansion/contraction of the gene families for the 17 selected plant species including *C. longa*, where *Arabidopsis thaliana* was used as an outgroup species. The "+" numbers (green colour) denote to the expanded gene family numbers for the selected species and ancestor nodes. The "-" numbers (red colour) denote to the contracted gene family numbers for the selected species and ancestor nodes. λ value for the clade formed by *C. longa* and *Musa acuminata* (species from Zingiberales plant order) was 0.011494, and for the rest of the species was 0.008130.

SUPPLEMENTARY NOTES

Supplementary Notes 1.

Sample collection and DNA Extraction

The plant sample was collected from an agricultural farm located in Bhopal, India (23.2280252°N 77.2088987°E). The plant was brought to lab and processed immediately. The leaves were used for DNA extraction using Carlson lysis buffer [100mM Tris HCl, 2% CTAB (Cetyl Trimethyl Ammonium Bromide), 1.4M NaCl, 1% PEG 8000, 20 mM EDTA pH 9.5] supplemented with β-mercaptoethanol (2.5 µl for each ml of buffer) for lysis[7]. Before adding the sample to buffer, it was allowed to pre-heat at 65˚C for 30 mins. The homogenized powder of leaves was added to the pre-heated buffer (1 ml) which was supplemented with 2 µl of RNase A (20 mg/ml) and 25 µl of Proteinase K (20 µL/mL). The tubes were given 1 hour of incubation at 65˚C with in-between mixing. In order to obtain high molecular DNA, the tubes were mixed by inverting the tubes in all the steps. All the centrifugation steps were performed at 5,000xg at 4˚C. After cooling the tubes at room temperature, 1 ml of chloroform was added and centrifuged for 15 mins. The aqueous layer formed was transferred to new centrifuge tube, ice-cold Isopropanol (0.7x volume) was added and followed by an overnight incubation at -20˚C in order to facilitate DNA precipitation. The tubes were centrifuged for 45 mins to pellet down the precipitated DNA. The DNA pellet was dissolved in 500 µl of G2 buffer (QiaAmp Blood and Cell culture Kit) by incubating at 50˚C for 15 mins. The Genomic tip 20 was equilibrated and the dissolved DNA was allowed to pass through it. Here, multiple tubes (up to 2 ml of G2 buffer) were loaded to single genomic tip 20 in order to increase the yield. Buffers were allowed to pass through the Genomic tip 20 via gravity flow. 1 ml of QC buffer was used thrice for washing the column and then DNA elution was done in 1 ml of QF buffer (pre-heated at 55˚C). The DNA was precipitated with 0.7X volume of ice-cold isopropanol and facilitated by an overnight incubation at -20˚C. The DNA was centrifuged for 30 mins at 4˚C to pellet it down. The DNA pellet was washed with 1 ml of ice-cold 70% ethanol, air dried to remove the residual ethanol and finally eluted in 50 µl of nuclease free water. For Nanopore sequencing, the extracted DNA was further purified using Ampure XP Magnetic beads (Beckman Coulter, Brea, CA USA). The NanoDrop™8000 Spectrophotometer (ThermoFisherScientific, USA) and 0.8-1% agarose gel electrophoresis, and Qubit 2.0 Fluorometer using Qubit dsDNA BR assay kit (Invitrogen, USA) were used to assess the quality and quantity of extracted DNA, respectively.

Species Identification

Species identification was done by using primers for a nuclear gene (Internal Transcribed Spacer ITS) and a chloroplast gene (Maturase K). The forward and reverse primers for complete ITS amplification were 5’-

TCCGTAGGTGAACCTGCGG-3′ and 5′-TCCTCCGCTTATTGATATGC-3′, respectively. For ITS2 gene amplification the primer set used was 5′-GCATCGATGAAGAACGCAGC-3′ and 5′-TCCTCCGCTTATTGATATGC-3′ as forward and reverse primers, respectively. The PCR programme ran on Veriti 96 well thermal cycler (Applied Biosystems) for ITS gene amplification was 94˚C for 3 mins, 35 cycles of 94˚C for 1 min, 55˚C for 1 min and 72˚C for 2.5 mins and 72˚C for 10 mins. Similarly, the primer set for Maturase K (MatK) included 5′-CGATCTATTCATTCAATATTTC-3′ as forward primer and 5′-TCTAGCACACGAAAGTCGAAGT-3′ as reverse primer. The PCR programme ran for MatK was 95˚C for 3 mins, 35 cycles of 95˚C for 30 sec, 50˚C for 3 mins and 72˚C for 1:15 min and final extension at 72˚C for 7 mins. The amplification was assessed on 2X agarose gel electrophoresis. The PCR product was purified and sequenced at in-house Sanger sequencing facility. The species was confirmed as *Curcuma longa* by checking the sequence identity and alignment with NCBI database using BLASTN.

Transcriptome Extraction

For RNA extraction, the powdered leaves (50-100mg) were added to 1 ml of TriZol reagent (Invitrogen, USA) and shaken for 5 mins. For ensuring complete dissociation of nucleoprotein complexes, the tubes were incubated at room temperature for 5 mins. To each tube, 200 ul of chloroform was added and vortexed for 15 seconds followed by incubation of 10 mins at room temperature (RT). In order to separate phases, the tubes were centrifuged at 12,000xg for 15 mins at 4˚C. The upper aqueous layer was transferred to a new tube where the RNA was allowed to precipitate with 500 ul of ice-cold isopropanol by incubating at RT for 5-10 mins. The RNA was pelleted by centrifuging at 12,000xg for 10 mins at 4˚C. Washing of RNA pellet was done with 1 ml of 75% ethanol. The pellet was dried by keeping it at 37˚C for 30 mins. The RNA pellet was resuspended in 30ul of nuclease free water by incubating it at 55-60˚C for 10-15 mins[8]. The quality and quantity of RNA was assessed by NanoDrop™8000 Spectrophotometer (ThermoFisherScientific, USA) and Qubit 2.0 Fluorometer using Qubit ssRNA HS assay kit (Invitrogen, USA), respectively.

Genomic and Transcriptomic Sequencing

For genomic sequencing the DNA library was prepared using Chromium Controller instrument, Chromium™ Genome Library & Gel Bead Kit v2 (10x Genomics, CA, USA) by following the manufacturer's instructions. The transcriptomic library was prepared with TruSeq Stranded Total RNA Library Preparation kit (Illumina, Inc., United States) by following the manufacturer's protocol with Ribo-Zero workflow. The quality of libraries was evaluated on Agilent 2200 TapeStation using High Sensitivity D1000 ScreenTape (Agilent, Santa Clara, CA). Both the libraries (Genomic and transcriptomic) were sequenced on Novaseq 6000 (Illumina, Inc., United States) generating 150 base pair paired-end reads. The Nanopore libraries

were constructed using SQK-LSK109 library preparation kit and following Genomic DNA by Ligation (SQK-LSK109) protocol of Oxford Nanopore Technologies (ONT, UK) with a few modifications such as starting DNA material was taken as 1.5 µg and 15-20 mins incubation for adapter ligation. The prepared libraries were loaded on FLO-MIN106 flowcells and sequenced on MinION Mk1b instrument (ONT, UK).

Supplementary Notes 2.

Genome size estimation

Barcode sequences were trimmed from raw 10x Genomics linked reads using a set of python scripts (https://github.com/ucdavis-bioinformatics/proc10xG). Barcodes were extracted from all paired-end linked reads using process_10xReads.py script with default parameters and irrespective of presence of valid gem barcodes. Reads were then filtered based on barcode status, using filter_10xReads.py script.

Genome size was estimated using a k-mer count distribution method implemented in SGA-preqc, that removes error prone k-mers with low occurrence count[9] . First, sga preprocess was used in paired-end mode with filtered linked reads; then the preprocessed reads were indexed with 'ropebwt' indexing algorithm and '--no-reverse' option; finally sga preqc was run with default settings to estimate the genome size.

Genome assembly and polishing

A total of 631.11 million 10x Genomics linked reads, corresponding to ~82.4X sequencing coverage, was used without any pre-processing, for *de novo* assembly of *C. longa* genome using Supernova v2.1.1 with maxreads=all option and Supernova mkoutput in 'pseudohap' style was used to generate the haplotype-phased fasta assembly file[10]. Barcodes from the raw reads were processed using Longranger basic v2.2.2 (https://support.10xgenomics.com/genome-exome/software/pipelines/latest/installation), to use in further assembly post-processing purpose. Tigmint v1.1.2 was used for correcting the mis-assemblies using the long range information resided within the linked reads[11]. First, the assembled genome was indexed and barcode-processed linked reads were mapped using BWA-MEM and samtools v1.9 was used to generate the ".bam" file[12,13]. This ".bam" file was used by tigmint-molecule to generate the ".bed" file that is further used by tigmint-cut to cut the mis-assembled regions and generate the corrected assembly.

For long reads-based assembly, 47.2 Gb of adapter-processed Nanopore data was used for *de novo* genome assembly using Flye v2.4.2 with default parameters[14]. Pilon v1.23 was used for correction of local mis-assemblies, indels, or errorneous bases in three iterations. Before each iteration, the barcode-processed 10x Genomics linked reads were mapped to the indexed genome using BWA-MEM[12], and the

alignments were converted to ".bam" format using samtools v1.9[13], which were then used in Pilon analysis.

Both the assemblies obtained from Supernova and Flye were scaffolded using barcode-processed 10x Genomics linked reads, quality-filtered RNA-Seq reads from this study, and adapter-processed Nanopore long reads (>20 Kb). For first round of scaffolding, linked reads were mapped to the corrected genome assembly using Longranger align v2.2.2 (https://support.10xgenomics.com/genome-exome/software/pipelines/latest/installation) and samtools v1.9 was used to generate the ".bam" file[13]. Using this ".bam" file, combination of ARCS v1.1.1 and LINKS v1.8.6 was used with default parameters, to generate a more contiguous assembly[15,16]. For further scaffolding, AGOUTI v0.3.3 was used with the filtered paired-end RNA-Seq reads that were required for *de novo* transcriptome assembly[17]. These paired-end RNA-Seq reads were mapped to previously scaffolded genome using BWA-MEM and samtools v1.9 was used to generate the ".bam" file[12,13]. Using this ".bam" file and AUGUSTUS v3.2.3 derived ".gff3" file[18], AGOUTI was used to generate further scaffolded genome assembly[17]. Adapter-processed Nanopore long reads (>20 Kb) were also used for another round of scaffolding using LINKS v1.8.6, with default values of "-l", "-t" and "-a" parameters[16].

Sealer v2.1.5 was used to gap-close this scaffolded genome assembly with barcode-processed linked reads and k-mer values from 30 to 120 (with an interval of 10 bp) with a Bloom-filter size of 950 GB[19]. Further gap-closing was performed with the adapter-processed Nanopore long reads using LR_Gapcloser[20]. Barcode-processed linked reads were again mapped to this gap-closed genome assembly using BWA-MEM and samtools v1.9 was used to generate the ".bam" file[12,13]. Finally, Pilon v1.23 was used with this ".bam" file to polish the genome assembly and improve the assembly quality[21].

Transcriptome data processing

Trimmomatic v0.38 was used for pre-processing of RNA-Seq data[22]. Adapter trimming was performed with maximum 2 mismatches in a 16-base seed matching; 30 and 10 was used as palindrome clip threshold and simple clip threshold, respectively. Quality threshold was set to 20 for leading and trailing end trimming of a read. Sliding window trimming for the reads was performed if an average quality score for a 4-base window went under 20. All reads containing less than 60 bases were removed.

de novo transcriptome assembly of *C. longa* using data from this study, and other previous studies resulted in a total of 383,724,313 assembled bases (N50 value of 1,531 bp), which consisted of 426,484 transcripts.

Supplementary Notes 3.

Tandem Repeats identification

For tandem repeat identification on the final polished *C. longa* draft genome (contigs with length of ≥1,000 bp after scaffolding), Tandem Repeat Finder (TRF) v4.09 was used with the parameters as follows: matching weight = 2, mismatching penalty = 7, indel penalty = 7, match probability = 80%, indel probability = 10%, minimum alignment score = 50, and maximum period size = 2,000[23].

Identification of transfer RNAs (tRNAs)

tRNAscan-SE v2.0.5 was used for *de novo* prediction of tRNAs in final polished *C. longa* draft genome assembly (contigs with length of ≥1,000 bp after scaffolding) with default parameters[24]. A total of 2,066 tRNAs were predicted, which were further classified as follows:

tRNAs decoding Standard 20 AA: 1,826

Selenocysteine tRNAs (TCA): 0

Possible suppressor tRNAs (CTA,TTA,TCA): 3

tRNAs with undetermined/unknown isotypes: 22

Predicted pseudogenes: 215

Along with these, 96 tRNAs with introns were identified.

Identification of microRNAs

miRBase database was used for homology-based identification of hairpin miRNAs[25]. A total of 38,589 hairpin miRNAs were clustered using CD-HIT-EST v4.8.1 with 90% sequence identity, to generate 22,365 non-redundant sequences[26]. Using these sequences, BLASTN was used with parameters of 80% identity and e-value 1e-03, to identify the hairpin miRNAs in *C. longa* final draft genome assembly[27].

SUPPLEMENTARY REFERENCES

1. Sheeja, T. E., Deepa, K., Santhi, R. & Sasikumar, B. Comparative Transcriptome Analysis of Two Species of Curcuma Contrasting in a High-Value Compound Curcumin: Insights into Genetic Basis and Regulation of Biosynthesis. *Plant Mol. Biol. Report.* (2015) doi:10.1007/s11105-015-0878-6.

2. Annadurai, R. S. *et al.* De Novo Transcriptome Assembly (NGS) of Curcuma longa L. Rhizome Reveals Novel Transcripts Related to Anticancer and Antimalarial Terpenoids. *PLoS One* (2013) doi:10.1371/journal.pone.0056217.

3. Sahoo, A., Jena, S., Sahoo, S., Nayak, S. & Kar, B. Resequencing of Curcuma longa L. cv. Kedaram through transcriptome profiling reveals various novel transcripts. *Genomics Data* (2016) doi:10.1016/j.gdata.2016.08.010.

4. Sahoo, A., Kar, B., Sahoo, S., Ray, A. & Nayak, S. Transcriptome profiling of Curcuma longa L. cv. Suvarna. *Genomics Data* (2016) doi:10.1016/j.gdata.2016.09.001.

5. Matasci, N. *et al.* Data access for the 1,000 Plants (1KP) project. *GigaScience* (2014) doi:10.1186/2047-217X-3-17.

6. Ou, S., Chen, J. & Jiang, N. Assessing genome assembly quality using the LTR Assembly Index (LAI). *Nucleic Acids Res.* (2018) doi:10.1093/nar/gky730.

7. Sánchez-Hernández, C. & Gaytán-Oyarzún, J. C. Two mini-preparation protocols to DNA extraction from plants with high polysaccharide and secondary metabolites. *African J. Biotechnol.* (2006) doi:10.5897/AJB2006.000-5076.

8. Johnson, M. T. J. *et al.* Evaluating Methods for Isolating Total RNA and Predicting the Success of Sequencing Phylogenetically Diverse Plant Transcriptomes. *PLoS One* (2012) doi:10.1371/journal.pone.0050226.

9. Simpson, J. T. & Durbin, R. Efficient de novo assembly of large genomes using compressed data structures. *Genome Res.* (2012) doi:10.1101/gr.126953.111.

10. Weisenfeld, N. I., Kumar, V., Shah, P., Church, D. M. & Jaffe, D. B. Direct determination of diploid genome sequences. *Genome Res.* (2017) doi:10.1101/gr.214874.116.

11. Jackman, S. D. *et al.* Tigmint: Correcting assembly errors using linked reads from large molecules. *BMC Bioinformatics* (2018) doi:10.1186/s12859-018-2425-6.

12. Li, H. Aligning sequence reads, clone sequences and assembly contigs with BWA-MEM. **00**, 1–3 (2013).

13. Li, H. *et al.* The Sequence Alignment/Map format and SAMtools. *Bioinformatics* (2009) doi:10.1093/bioinformatics/btp352.

14. Kolmogorov, M., Yuan, J., Lin, Y. & Pevzner, P. A. Assembly of long, error-prone reads using repeat graphs. *Nat. Biotechnol.* (2019) doi:10.1038/s41587-019-0072-8.

15. Yeo, S., Coombe, L., Warren, R. L., Chu, J. & Birol, I. ARCS: Scaffolding genome drafts with linked reads. *Bioinformatics* (2018) doi:10.1093/bioinformatics/btx675.

16. Warren, R. L. *et al.* LINKS: Scalable, alignment-free scaffolding of draft genomes with long reads. *Gigascience* (2015) doi:10.1186/s13742-015-0076-3.

17. Zhang, S. V., Zhuo, L. & Hahn, M. W. AGOUTI: Improving genome assembly and annotation using transcriptome data. *Gigascience* (2016) doi:10.1186/s13742-016-0136-3.

18. Stanke, M. *et al.* AUGUSTUS: A b initio prediction of alternative transcripts. *Nucleic Acids Res.* (2006) doi:10.1093/nar/gkl200.

19. Paulino, D. *et al.* Sealer: A scalable gap-closing application for finishing draft genomes. *BMC Bioinformatics* (2015) doi:10.1186/s12859-015-0663-4.

20. Xu, G. C. *et al.* LR-Gapcloser: A tiling path-based gap closer that uses long reads to complete genome assembly. *Gigascience* (2018) doi:10.1093/gigascience/giy157.

21. Walker, B. J. *et al.* Pilon: An integrated tool for comprehensive microbial variant detection and genome assembly improvement. *PLoS One* (2014) doi:10.1371/journal.pone.0112963.

22. Bolger, A. M., Lohse, M. & Usadel, B. Trimmomatic: A flexible trimmer for Illumina sequence data. *Bioinformatics* (2014) doi:10.1093/bioinformatics/btu170.

23. Benson, G. Tandem repeats finder: A program to analyze DNA sequences. *Nucleic Acids Res.* (1999) doi:10.1093/nar/27.2.573.

24. Chan, P. P. & Lowe, T. M. tRNAscan-SE: Searching for tRNA genes in genomic sequences. in *Methods in Molecular Biology* (2019). doi:10.1007/978-1-4939-9173-0_1.

25. Griffiths-Jones, S., Saini, H. K., Van Dongen, S. & Enright, A. J. miRBase: Tools for microRNA genomics. *Nucleic Acids Res.* (2008) doi:10.1093/nar/gkm952.

26. Fu, L., Niu, B., Zhu, Z., Wu, S. & Li, W. CD-HIT: Accelerated for clustering the next-generation sequencing data. *Bioinformatics* (2012) doi:10.1093/bioinformatics/bts565.

27. Altschul, S. F., Gish, W., Miller, W., Myers, E. W. & Lipman, D. J. Basic local alignment search tool. *J. Mol. Biol.* (1990) doi:10.1016/S0022-2836(05)80360-2.
