# Supplementary material for: Genome sequencing of turmeric provides evolutionary insights into its medicinal properties
Source: Commun Biol. 2021 Oct 15;4:1193. doi: 10.1038/s42003-021-02720-y (PMC8521574; doi:10.1038/s42003-021-02720-y)
Supplement: Supplementary file 2 — Description of Additional Supplementary Files [file 42003_2021_2720_MOESM2_ESM.pdf]

Description of Additional Supplementary Files

File name: Supplementary Data 1.

Description: The details of C. longa genes showing multiple signs of adaptive evolution (MSA).
